# Dynamic patterns of gene expression match extracellular signals through push-pull regulation

**DOI:** 10.1371/journal.pgen.1011943

**Published:** 2025-11-19

**Authors:** Luis Fernando Montano-Gutierrez, Marc Sturrock, Iseabail L. Farquhar, Kevin Correia, Vahid Shahrezaei, Peter S. Swain

**Affiliations:** 1 School of Biological Sciences, University of Edinburgh, Edinburgh, United Kingdom; 2 Department of Physiology, Royal College of Surgeons in Ireland, Dublin, Ireland; 3 Department of Mathematics, Imperial College London, London, United Kingdom; Texas A&M University, UNITED STATES OF AMERICA

## Abstract

Cells can match gene expression to a range of a particular signal. For example, budding yeast expresses at least seven hexose-transporter (HXT) genes in different concentration ranges of extracellular glucose. Using time-lapse microscopy, microfluidics, dynamic glucose inputs, and mathematical modelling, we determine how this glucose matching of HXT expression occurs mechanistically. The glucose-sensing network generates a push-pull regulation using two pairs of regulators: rising glucose weakens, or “pulls”, repression via regulators Mth1 and Std1 while simultaneously strengthening, or “pushing”, repression via regulators Mig1 and Mig2; falling glucose reverses this push-pull. The regulators’ combined activity reports extracellular glucose. Cells match HXT expression to glucose because HXT promoters couple to the regulators in ways specific to low, medium, or high-affinity transporters. By rewiring transcription and using model-predicted perturbations, we demonstrate how an HXT encoding a medium-affinity transporter can respond as one encoding either a low- or a high-affinity transporter. Matching gene expression to a pattern of input is fundamental; we believe push-pull regulation to be widespread.

## Author summary

Cells not only express genes when an input signal passes a threshold concentration, but also for ranges of a signal’s concentration. We studied mechanistically how cells perform this matching using budding yeast, which expresses different glucose-transporter genes in different concentration ranges of extracellular glucose. Combining time-lapse microscopy and mathematical modelling, we demonstrated that the regulation is through two types of transcriptional regulators, one type whose nuclear concentration increases with extracellular glucose and another type whose nuclear concentration decreases with extracellular glucose. How genes couple to these regulators determines in which concentration range of glucose they express. The solution mirrors one in fly development, where the embryo matches expression to spatial locations also through two types of regulators, one whose concentration increases with distance from an embryo’s pole and one whose concentration decreases. Such push-pull regulation encodes in the regulators’ concentrations the value of a cue, such as the concentration of extracellular glucose for yeast and the intracellular location for the fruit fly embryo. We believe therefore that this solution to matching gene expression is commonplace.

## Introduction

Cells regulate gene expression to express genes opportunely. An iconic example is *Escherichia coli*’s *lac* operon, which expresses the genes for *lac* permease and other enzymes only when induced [15]. Intracellular allolactose has to reach a sufficiently high concentration before the *lac* repressor inactivates, enabling transcription [39]. At a population level, the net effect is a graded response that increases with the concentration of extracellular inducer [48]. Genomes however often encode not just one transporter for a particular nutrient, like *lac* permease, but multiple [25].

**Fig 1:**
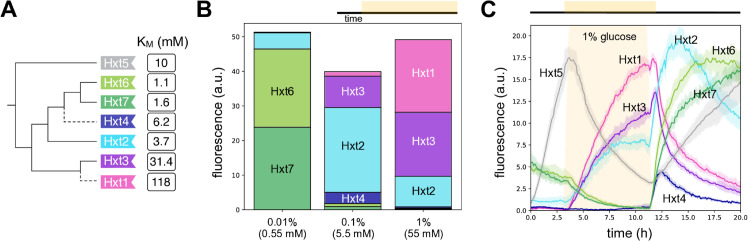
*HXT* expression depends on the affinity of the transporter encoded. **A** Budding yeast commonly express seven hexose transporters. Each transporter has a different affinity for glucose given by the reciprocal of its *K*_*M*_ [7,27,41]. We show the transporters’ phylogenetic relationships (S1 Text), with dashed lines indicating the whole genome duplication. **B** After 10 hours in constant concentrations of glucose, the most abundant Hxts have values of *K*_*M*_ that best match the glucose concentration. Individual bars are in Fig B panel E in S1 Text. We show the population-mean fluorescence per cell of strains with one *HXT* C-terminally tagged with GFP. Each bar is an average over at least two and at most five experimental replicates. We switched to glucose medium after cells had spent approximately 3 h in the microfluidic device and present data from 14 h into each experiment. **C** In dynamic glucose concentrations, the *HXT*s can have diverse behaviours, with some responding only to falling concentrations. We show the population-mean levels for cells growing in synthetic complete medium where glucose rose from zero to a concentration of 1% at approximately 3 hours, at a rate of 1.5% h^−1^, and fell to zero again at approximately 11 hours, at a rate of 2% h^−1^. Glucose’s presence is indicated by orange. Data are averages over two experimental replicates (Fig B panel F in S1 Text) and the shading shows standard errors of the mean. For both **B** and **C**, cells were at exponential phase in 2% galactose before being placed in the microfluidic device. Galactose induced expression of some *HXTs* leading to non-zero initial levels for Hxt2, Hxt6, and Hxt7.

The model eukaryote budding yeast, for example, has 18 genes encoding potential transporters of glucose [3]. Cells lacking *HXT1* through *HXT4* and *HXT6* through *HXT7* do not grow aerobically on glucose, but adding back any one re-enables growth [40]. Each of these *HXT*s is expressed in a particular range of glucose concentrations [8,19,28,31,34,60,61], and each Hxt transporter has a different affinity for glucose [27,41] (Fig 1A): Hxt1 and Hxt3 have a low affinity; Hxt2 and Hxt4 have a medium affinity; and Hxt6 and Hxt7 have a high affinity. Hxt5, although its regulation is distinct [57,58], has medium affinity too.

Analogous to shifting gears in a bicycle, cells express different *HXT*s as the extracellular glucose concentration changes [8,19,28,31,34,60,61]. Each transporter works by facilitating diffusion down a sugar concentration gradient and consequently likely has a rate-affinity trade-off [4,30]. Transporters with a high affinity for glucose necessarily have a low maximal rate of transport, and vice versa. Having a higher affinity makes the transporter-glucose complex more stable, both extra- and intracellularly, reducing the overall transport rate. Cells are thought to circumvent the trade-off by having multiple *HXT*s, switching *HXT* expression to maintain a high sugar influx despite changing extracellular glucose [30].

Although the structure of the genetic network regulating the *HXT*s is largely known [3,35,51], why it has that structure is less so. If cells are evading a trade-off in transport, as we postulate, the network must sense glucose and express those *HXT*s having affinities that match glucose’s current concentration: low-affinity transporters in high extracellular glucose and high-affinity in low.

We therefore set out to understand mechanistically how budding yeast’s *HXT* network functions, with the aim of answering the more general question of how cells might match gene expression to a concentration range of a regulatory molecule, rather than have expression proportional to its concentration. Our approach was to employ microfluidics to control extracellular glucose and time-lapse microscopy to follow expression of fluorescently tagged *HXT* genes. Using dynamic inputs, deletion strains, mathematical modelling, and statistical inference, we show that two pairs of repressors interchange their activities, one pair active and the other inactive, as the glucose concentration falls or rises and that the *HXT* promoters match their expression with glucose through how they couple to these repressor pairs.

## Results

### Cells match *HXT*s by their affinity to constant glucose concentrations

If a rate-affinity trade-off makes each Hxt transporter have a range of glucose concentrations for which its import flux is greater than any other Hxt [30], then, naively assuming no other constraints, we might expect cells in constant glucose to express only one, the optimal, *HXT*. Therefore we used microfluidics to grow cells close to steady state in flowing media of different glucose concentrations. In our ALCATRAS devices [6] (Fig A in S1 Text), cells reside in individual “jails”, made of a transparent polymer, and the medium washes away their daughters. The constant flow of medium and the limited number of cells, typically one or two per microscopic jail, prevents cellular growth substantially altering extracellular glucose concentrations. We used Green Fluorescent Protein (GFP) to tag *HXT1-7* and measured the mean fluorescence of each cell over time, processing the resulting time-lapse data automatically [1]. To compare quantitatively from experiment to experiment, we normalised the fluorescence data by the mean fluorescence in the absence of glucose of a control strain (Fig B panels A-D in S1 Text).

After 10 hours of growth in constant glucose, we find that multiple transporters are still co-expressed for all three concentrations of glucose tested (Fig 1B). Nevertheless, if we consider three classes of transporters (Fig 1A): a high-affinity class comprising Hxt6 and Hxt7, a medium-affinity comprising Hxt2 and Hxt4, and a low-affinity comprising Hxt1 and Hxt3, then at this level of classes the results conform with the predictions. The affinities of the most highly expressed transporters match the glucose concentration: in low 0.01% glucose, the high-affinity Hxt6 and Hxt7; in medium 0.1% glucose, the medium-affinity Hxt2; and in high 1% glucose, the low-affinity Hxt1 and Hxt3. The second most highly expressed transporter is always from a neighbouring class: medium-affinity Hxt2 in low glucose; low-affinity Hxt3 in medium glucose, and medium-affinity Hxt2 in high glucose. We did not report data for Hxt5 because its levels did not reach steady state: they rose in the glucose-free period of the experiment and then continuously fell but only slowly and at a rate weakly dependent on the glucose concentration.

Although the results of Fig 1B suggest that cells predominately regulate the *HXT*s to rapidly import the glucose currently available, they also suggest that cells might too prepare for future changes in glucose [24,42]. We therefore decided to determine how dynamic glucose inputs affected their response.

### The *HXT*s’ response to changing glucose is diverse

We submitted cells to a rising, constant, and then falling glucose concentration, switching from synthetic complete medium without glucose into eight hours of synthetic complete medium with constant glucose and then back to synthetic complete medium without glucose. For both switches, the glucose concentration changed over approximately 15 minutes. By switching in and out of a glucose-free medium and by pre-growing cells in pyruvate, a gluconeogenic carbon source, we ensured that the only response to glucose is the one generated by the switch in media.

We observed a remarkable diversity of behaviour (Fig 1C). There was a dependence of gene expression on the rate of change of extracellular glucose, with the response differing if glucose rose or fell. Levels of the medium-affinity Hxt4 and the high-affinity Hxt6 and Hxt7 spiked but only in decreasing glucose; levels of the medium-affinity transporter Hxt2 spiked when glucose began to both increase and decrease; levels of Hxt5 appeared to increase exclusively in the absence of glucose, consistent with earlier work [7]; and just the low-affinity transporters Hxt1 and Hxt3 behaved as conventionally expected — their levels increased only once the glucose concentration was sufficiently high. These behaviours held too at the single-cell level: we detected no distinct sub-populations (Fig C in S1 Text).

### Characterising and modelling the behaviour of *HXT4*

To determine if these responses are consistent with the known structure of the genetic network regulating the *HXT*s, we developed a mathematical model and compared its predictions to Fig 1 and to data generated using strains missing components of the network.

We decided to focus initially on the regulation of *HXT4*, which has similar, if more extreme, behaviour to *HXT2*, *HXT6*, and *HXT7*. All of these transporters responded differently to rising and falling glucose (Fig 1C), implying that more than a threshold concentration of extracellular glucose controls their expression. In our earlier work [31], we found too that expression from *HXT4-GFP* was the most responsive of these *HXT*s to deletions of regulatory genes. We verified that Hxt4’s spiking as glucose falls (Fig 1C) is not because of a serendipitously timed delay, seeing similar spiking in an experiment with a pulse of glucose lasting half as long (Fig D panel A in S1 Text). We also confirmed that it is transcriptional control that restricts the induction of the response to falling glucose, using a strain with GFP driven by the promoter of *HXT4*. Like *HXT4-GFP*, this strain’s fluorescence was minimal during 1% glucose and spiked only when glucose fell, although less sharply (Fig D panel B in S1 Text).

**Fig 2:**
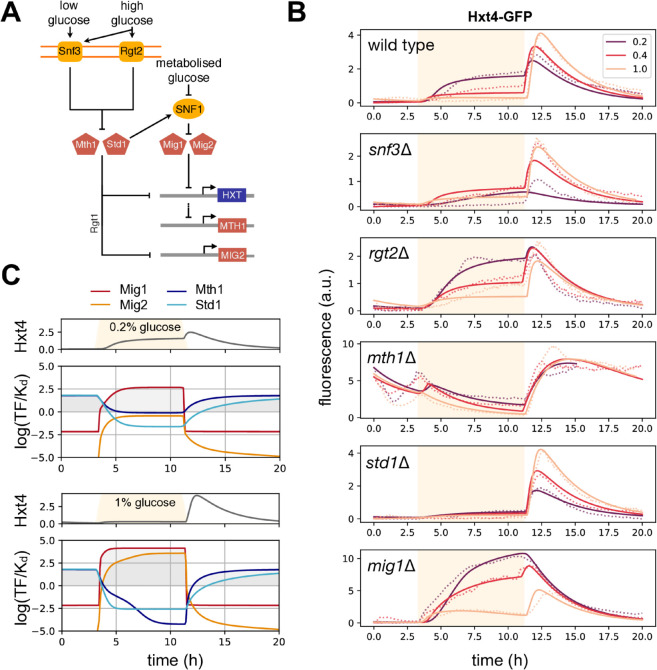
By developing and calibrating a mathematical model of the Snf3-Rgt2 network, we can quantitatively predict how *HXT4*’s repression changes with extracellular glucose. **A** Two pairs of repressors regulate the *HXT* genes. High glucose inactivates Mth1 and Std1, via the sensors Snf3 and Rgt2. Mth1 and Std1 are co-repressors, binding at promoters already bound by the transcription factor Rgt1. Low glucose inactivates Mig1 and Mig2. Low glucose activates SNF1 kinase, which phosphorylates and inactivates Mig1. Low glucose inactivates Mig2 by repressing *MIG2* transcription via Mth1 and Std1 and, in our model, increasing Mig2’s degradation. Mig1 and Mig2 repress *HXT*s and *MTH1*. **B** We used a systematic microfluidics-based study of Hxt4-GFP combined with mathematical modelling to determine how cells regulate *HXT4*. We exposed the wild-type and five deletion strains to dynamic glucose inputs that rose from zero to either 0.2%, 0.4%, or 1% glucose and then fell back to zero (orange shading). Dotted lines show the measured population-mean fluorescence per cell, averaged over at least two replicates; full lines show best-fit predictions from a mathematical model of **A**. Notice the scales of the *y*-axes for both the *mth1Δ* and *mig1Δ* strains. **C** The model predicts a spike of *HXT4* expression because Mig1 and Mig2 respond faster to changing glucose than Mth1 and Std1. Using the best-fit parameters, we plotted the log_2_ of the predicted level of active repressors scaled by their inferred dissociation constants of binding to the *HXT4* promoter. They repress when these variables are positive. Comparing glucose concentrations, *MIG2* induces sufficiently in 1% glucose to help lower the levels of Hxt4.

To develop our mathematical model, we started with the Snf3-Rgt2 network [33], which has *HXT* expression as its primary output. On sensing glucose, the network inactivates two co-repressors of the *HXT* genes, Mth1 and Std1. To do so the network use a low-affinity sensor for glucose, Rgt2, a high-affinity sensor, Snf3, and a transcriptional regulator, Rgt1 [33] (Fig 2A). If Rgt1 forms a complex at a *HXT* promoter with at least one of Mth1 and Std1 [22], it inhibits expression [34,38]. In the presence of glucose, Snf3 or Rgt2 or both sensors together cause inactivation of the co-repressors Mth1 and Std1 [51]. Cells inactivate Mth1 using degradation [10]. Although in glucose both Mth1 and Std1 are actively degraded, only levels of Mth1 decrease because of compensatory expression from *STD1* [18,44]. Cells inactivate Std1 instead using condensation: in sufficiently high glucose, Std1 condenses into cytoplasmic granules [50]. The network also regulates a gene for another repressor, *MIG2* [18], whose protein product inhibits *HXT* expression.

We also included the SNF1 kinase complex, budding yeast’s AMP kinase [5] (Fig 2A). SNF1 responds to low intracellular glucose [5] and regulates the *HXT*s through another repressor Mig1 [18]. Std1 stimulates SNF1’s activity [20,50], potentially directly [14,50,54]. Active SNF1 phosphorylates Mig1 [56], which then exits the nucleus, enabling *HXT* expression.

Our model focused on the four regulators. We let *MTH1* be repressed by Mig1 and Mig2 [18] and its protein Mth1 be degraded both at a constant rate and through Snf3 and Rgt2 at a rate that increases with extracellular glucose. We modelled nuclear Std1 assuming total levels of Std1 are constant [44]; Std1 is exported from the nucleus both at a constant rate and through Snf3 and Rgt2 at a rate that increases with extracellular glucose. We modelled nuclear Mig1 similarly assuming total levels of Mig1 are constant [2]; Mig1 is exported from the nucleus both at a constant rate and at a rate proportional to SNF1’s activity, which decreases with extracellular glucose and is an increasing function of Std1. Lastly, we let *MIG2* be transcriptionally repressed by Mth1 and Std1 and allowed the rate of degradation of its protein Mig2 to be a decreasing function of intracellular glucose. We assumed that Rgt1 is always bound to the *HXT* promoters, so that nuclear Mth1 and Std1 can also always bind. Although protein kinase A, the second major carbon-sensing kinase in yeast [5], phosphorylates Rgt1 when Rgt1 is free of Mth1 and Std1 [17], we omitted this behaviour because it appears to boost already active *HXT* expression [17].

We let all four repressors regulate *HXT4*. Comparing the response of GFP expressed from the *HXT4* promoter to GFP-tagged Hxt4 (Fig D panel B in S1 Text), we let Hxt4’s degradation rate decrease with the glucose concentration, so that Hxt4 degrades more rapidly when glucose is absent [13]. We found that a similar glucose-dependent degradation of Mig2 improved the comparison with the data and, as mentioned, included it in the model. Finally, we assumed minimal intracellular glucose unless extracellular glucose is present, and then we used a logistic function to describe the rise of intracellular glucose as the Hxts transport glucose into the cell. This logistic function’s growth rate, describing the rate of increase of intracellular glucose, was another model parameter, which we allowed to vary for different extracellular glucose concentrations and for the deletion mutants.

The net effect of the regulation captured by the model is that while rising glucose weakens repression through Mth1 and Std1, it strengthens repression through Mig1 and Mig2. Falling glucose reverts the response, strengthening repression through Mth1 and Std1 while weakening repression through Mig1 and Mig2. Glucose activates Mig1 through a double negative interaction, inhibiting SNF1, which inhibits Mig1. Glucose activates Mig2 more directly, by weakening *MIG2*’s repression by Mth1 and Std1.

In our experiments we measured the response of *HXT4-GFP* in the wild-type strain and five gene-deletion mutants in three different inputs of extracellular glucose (Fig 2B). We deleted, one at a time, the sensors *SNF3* and *RGT2* and the repressors *MTH1*, *STD1*, and *MIG1*, generating only the *mig1Δ* strain because Mig2 typically acts redundantly with Mig1 [59]. The wild-type response decreased in glucose with an increasing glucose concentration, but spiked higher when glucose fell (Fig 2B), with its behaviour in 0.2% glucose qualitatively similar to *HXT2-GFP* in 1% glucose (Fig 1C). Each mutant had distinct responses, with much higher expression in the absence of glucose for the *mth1Δ* strain and in the presence of glucose for the *mig1Δ* strain. Deleting the *STD1* co-repressor counter-intuitively reduced expression in glucose.

### A mechanistic explanation of *HXT4* expression

The model reproduced the observed behaviour, both for the wild type and mutants (Fig 2B). Using numerical optimisation (S1 Text), we fit the model’s parameters to the data of Fig 2B and to the time-series generating Fig 1B (Fig E panel A in S1 Text), generating tens of parameter sets consistent with the data. This fitting revealed two potential mechanisms, and it is helpful to consider the low expression of *HXT4* in the *std1Δ* strain in glucose to understand their difference. One mechanism is for Std1 to activate SNF1 (Fig 2A). Then deleting *STD1* hyperactivates Mig1 via a less active SNF1, and Mig1 represses *HXT4* in glucose. The other mechanism is for Std1 to strongly repress *MIG2*. Then deleting *STD1*, hyperactivates Mig2 and so represses *HXT4*. We selected a parameter set (S1 Text and Fig F in S1 Text) where Std1 predominately activated SNF1: this class of solutions had Std1 active in low glucose, consistent with glucose inducing *STD1* and repressing *MTH1* [18] and with our earlier work [31]. With these parameters, Mth1 dominates repression of *MIG2*, in a feedforward loop [21].

With the model, we could predict which repressors actively regulated *HXT4* (Fig 2C and Fig E panel B in S1 Text). In the absence of glucose, Mth1 and Std1 repress *HXT4*; in the presence of glucose, it is Mig1 and, as the glucose concentration rises, Mig2. The model predicts further that the lower expression in the *snf3Δ* strain is from more active Mth1 and Std1 and that the higher expression in the *rgt2Δ* strain is from less active Mig1. Rgt2 preferentially inactivates Std1 over Mth1 in the model whereas Snf3 inactivates both similarly. Deleting Rgt2 therefore causes more active Std1 and so more active SNF1 (Fig 2A), giving less active Mig1.

A difference in time scales generates the distinct responses in rising and falling glucose, including the spike in Hxt4 levels, with Mig1 and Mig2 responding quicker than Mth1 and Std1. In rising glucose, Mig1 and Mig2 activate faster than Mth1 and Std1 deactivate, inhibiting expression. In falling glucose, Mig1 and Mig2 inactivate faster than Mth1 and Std1 reactivate, creating a window where expression can occur (Fig 2C). For example, Mig1 and Mig2 responded on average over twice as fast both in rising and falling glucose in the 1% simulations.

### Inferring the regulation of the other *HXT*s

To address our initial question of how the cell matches *HXT* expression to extracellular glucose, we exploited the model of *HXT4*, taking its signalling component, replacing the *HXT4* promoter with a new one, and inferring this promoter’s structure for each *HXT* from the data of Fig 1. Assuming that at least one repressor binds and that at most all four do, there are 15 possible promoter structures. Previously we used plate readers to compare with wild type the levels of the Hxt-GFPs for *mth1Δ* and *std1Δ* mutants [31], and we used this data here to restrict *a priori* the number of possible models for each *HXT* (Fig G in S1 Text). For example, in that data the *std1Δ* mutant deviated substantially from the wild type for Hxt1- and Hxt3-GFP. We therefore required that all promoter models for *pHXT1* and *pHXT3* included Std1.

With these *a priori* choices, we used approximate Bayesian computation [23,55] to infer the posterior probability of each promoter structure, as well as parameters specific to an Hxt: its maximal rate of synthesis and degradation, and the dissociation constants and Hill numbers of the repressors binding to its promoter. We fixed all other parameters to the values inferred from the *HXT4* experiments (Fig 2B) and used the time-series data that generated Fig 1B and the data in Fig 1C for our inference (Fig H in S1 Text). We excluded *pHXT5* because it is not regulated by the Snf3-Rgt2 network [7] (Fig G panel B in S1 Text).

**Fig 3:**
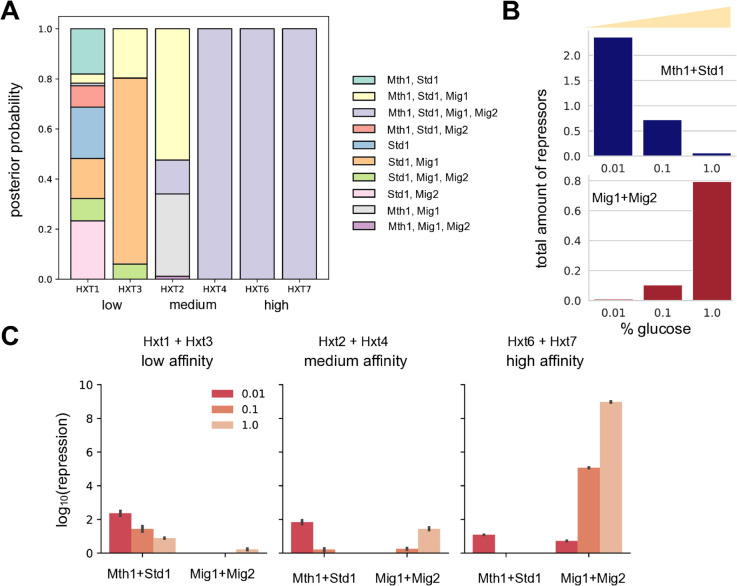
Mathematical modelling predicts that the promoters of transporters with similar glucose affinities have similar regulation. **A** We inferred the regulatory structure of each *HXT* by comparing multiple different promoter models with the data of Fig 1 using approximate Bayesian computation. All models included the signalling component of the *HXT4* model. We show the posterior probabilities of different promoters, defined by the particular repressors they bind (legend). For example, the most probable structure for *HXT3* is regulation by Std1 and Mig1. **B** The *HXT4* model predicts how the total steady-state level of active Mth1 and Std1 decreases as glucose rises while the total level of nuclear Mig1 and Mig2 increases. **C** The average steady-state repression for *HXT*s grouped by their affinities is distinct. Fig I in S1 Text gives the individual results. Glucose concentrations are percentages (g/100 mL). Errors are 95% confidence intervals found by bootstrapping from 100 samples of parameter values from each *HXT*’s posterior distribution over promoter models.

For *HXT6* and *HXT7*, we inferred that only one promoter model is compatible with the data, but for the other *HXT*s multiple models were possible, although usually one was unambiguously most probable (Fig 3A). Examining the models selected, we can predict that all four repressors regulate *HXT6* and *HXT7*, that at least Mth1 and Mig1 regulate *HXT2*, and that at least Std1 and Mig1 regulate *HXT3*. For *HXT1*, four models had similar posterior probabilities, and *a priori* all included Std1, implying that provided Std1 is present any other repressor or pair of repressors suffices.

### Modelling suggests that expression matches glucose through distinct coupling to the repressors

To interpret these results, we shifted scale, considering the combined effects of Mth1 and Std1 and of Mig1 and Mig2. We believe that the data of Fig 1 are rich enough to capture behaviour at this scale correctly, but less so at the scale of the individual repressors.

We examined the predicted regulation of each *HXT* in the constant glucose concentrations of Fig 1B: low (0.01%), medium (0.1%), and high (1%). When glucose rises, the predicted amount of active Mth1 and Std1 decreases and that of Mig1 and Mig2 increases (Fig 3B), as expected [2,18,44,50]. Using the posterior probabilities (Fig 3A), we sampled from the models for each *HXT* and for each sample found the repression exerted by both pairs of repressors. To measure repression, we used the weight by which the repressor pair diminished expression in the model (see the transcriptional term in Eq 7 in S1 Text): for *R*_*i*_ molecules of repressor, a promoter dissociation constant *K*_*i*_, and a Hill number *n*_*i*_, the repression for a pair of repressors is $(R_{1}/K_{1}){\;}^{n_{1}}+(R_{2}/K_{2}){\;}^{n_{2}}$. Although we estimated the mean repression for each *HXT* (Fig I in S1 Text), the results are clearer at a higher scale, averaging the mean repression for the two low-affinity transporters, Hxt1 and Hxt3, the two medium-affinity transporters, Hxt2 and Hxt4, and the two high-affinity transporters, Hxt6 and Hxt7.

The predicted regulation is distinct for these collective transporters and determined by their affinity (Fig 3C). The low-affinity transporters respond principally to Mth1 and Std1, expressing in high glucose (Fig 1B) because the repression from these two repressors decreases as glucose rises. In contrast, the high-affinity transporters respond principally to Mig1 and Mig2, expressing in low glucose (Fig 1B) because repression from these repressors increases as glucose rises. To express in medium glucose, the medium-affinity transporters respond to both pairs of repressors, repressed in low glucose by Mth1 and Std1 and in high glucose by Mig1 and Mig2. Consistently, repression by Mth1 and Std1 of the medium-affinity transporters in low glucose is higher than their repression of the high-affinity transporters, and repression by Mig1 and Mig2 of the medium-affinity transporters in high glucose is higher than their repression of the low-affinity transporters.

**Fig 4:**
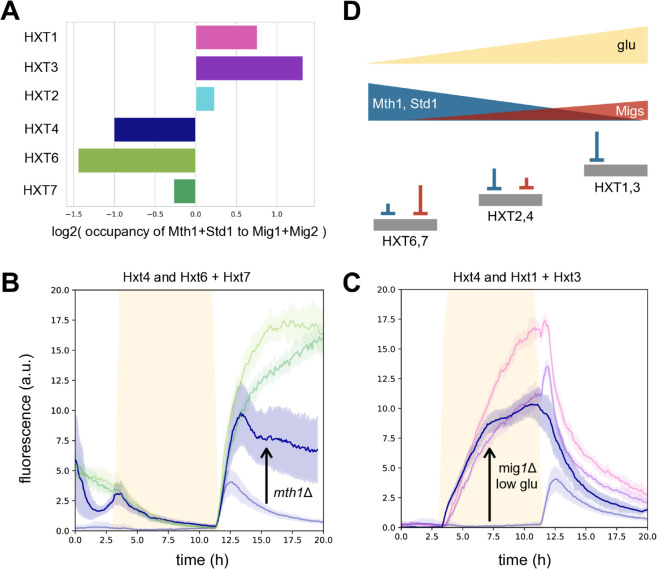
By coupling appropriately to two pairs of repressors that respond to glucose in opposite ways, *HXT*s express in the glucose concentration that matches their transporter’s affinity. **A** Estimating the occupancy of Rgt1, to which Mth1 and Std1 bind, and of Mig1 and Mig2 using SwissRegulon’s predicted binding sites [36] shows that the expected bias of the *HXT* promoters for the two pairs of repressors is consistent with the predictions of Fig 3C. **B** Reducing repression of *HXT4* by Mth1 and Std1 through deleting *MTH1* causes Hxt4 (blue) to mimic the high-affinity transporters Hxt6 and Hxt7 (greens). Data are from Figs 1C & 2B (both 1% glucose). **C** Reducing repression of *HXT4* by Mig1 through deleting *MIG1* and by Mig2 through lowering the glucose concentration to decrease *MIG2*’s induction causes Hxt4 (blue) to mimic the low-affinity transporters Hxt1 and Hxt3 (purples). Data are from Fig 1C (1% glucose) and Fig 2B (0.2% glucose). **D** Cells use a push-pull system of repressors to match *HXT* expression to glucose by the affinity of the transporters they encode. The signalling network causes two pairs of repressors to respond in opposite ways to glucose. Promoters for high-affinity transporters express in low glucose by coupling to Mig1 and Mig2; promoters for medium-affinity transporters express in medium glucose by coupling to both repressor pairs; and promoters for low-affinity transporters express in high glucose by coupling to Mth1 and Std1. We indicate the extent of repression schematically by the length of the inverted T, following the results of Fig 3C.

We used bioinformatics to determine if these predictions are consistent with the sequences of the *HXT* promoters. Recall that Mth1 and Std1 do not bind directly to the DNA but to the transcriptional activator Rgt1 [22]. We therefore used SwissRegulon [36] to find the potential binding sites of Rgt1, Mig1, and Mig2 for each *HXT* promoter. SwissRegulon returns a posterior probability for each binding site allowing us to quantify the total probability of binding though estimating the occupancy of the transcription factors at the promoters assuming their concentrations are low (S1 Text). Grouping Mig1 and Mig2 together, we compared the predicted occupancy of Rgt1, and so of Mth1 and Std1, to the predicted occupancy of Mig1 and Mig2 (Fig 4A). As expected (Fig 3C), the promoters for high-affinity transporters favoured binding of Mig1 and Mig2 over Mth1 and Std1, and the promoters for low-affinity transporters promoters favoured the opposite. Those for medium-affinity transporters were, again as expected, intermediate: *pHXT2* having a bias of the same sign as *pHXT1* and *pHXT3* but not as extreme, and *pHXT4* having a bias of the same sign as *pHXT6* and *pHXT7* but not as extreme as *pHXT6*. We suspect the discrepancy between *pHXT6* and *pHXT7*, which contradicts their similar levels of expression (Fig 1), is because SwissRegulon uses the adjacent, upstream intergenic region as the promoter for each gene. The intergenic region of *HXT7* is more than twice as long as that for *HXT6* (Fig J in S1 Text), increasing the likelihood of false positives if *HXT7*’s promoter is smaller than this region.

To verify these predictions we re-examined the responses of the deletion mutants. We expected that a medium-affinity *HXT*, such as *HXT4*, should respond like a high-affinity *HXT* if we weakened the binding of Mth1 and Std1 to its promoter whereas it should respond like a low-affinity *HXT* if we weakened the binding of Mig1 and Mig2 (Fig 3C). Although mutating *HXT4*’s promoter would modify binding, which mutations to make are unclear because Mth1 and Std1 do not themselves bind DNA but the transcription factor Rgt1. Rgt1’s behaviour is complex; it may repress transcription in one state and activate transcription in another [17,32,43]. Instead we re-considered the strains with deleted repressor genes (Fig 2B). Deleting *MTH1* should weaken repression by Mth1 and Std1. In contrast, our modelling predicted that Std1 contributed little to *HXT4*’s repression in high glucose consistent with *STD1*’s deletion leaving Hxt4’s behaviour almost unchanged (Fig 2B). As expected (Fig 3C), the behaviour of Hxt4 in the *mth1Δ* strain mimicked the high-affinity transporters Hxt6 and Hxt7 (Fig 4B). Deleting *MIG1* and reducing *MIG2* expression by lowering the glucose concentration should weaken repression by Mig1 and Mig2. Again as expected (Fig 3C), the behaviour of Hxt4 in low glucose then mimicked the low-affinity transporters Hxt1 and Hxt3 in high glucose (Fig 4C).

## Discussion

To understand how cells match gene expression to a concentration range of an input, we have studied budding yeast’s *HXT* network with time-lapse microscopy and mathematical modelling. We first focused on *HXT4*, fitting a model to dynamic time-series responses of the wild-type and mutant strains. This model included gene regulation and glucose-sensing by Snf3 and Rgt2 and by the SNF1 kinase (Fig 2). Next we extended the model to the other *HXT*s by inferring the likely structure of their promoters from wild-type data (Fig 3). Together these models suggested how the network functions, and we tested this mechanism (Fig 4) both with a bioinformatic analysis of the promoters’ DNA sequences and with mutants and perturbations that we expected should cause one *HXT* to behave like another.

On a broad scale, we showed that yeast cells express the *HXT*s so that the affinity of their transporters matches the concentration of extracellular glucose (Fig 1B). They do so using two pairs of repressors that we propose work together as a push-pull system. In rising glucose, cells ‘pull’ repression by Mth1 and Std1 and ‘push’ repression by Mig1 and Mig2 (Fig 4D); in falling glucose, they do the opposite, pulling repression by Mig1 and Mig2 and pushing repression by Mth1 and Std1. The nuclear concentrations of active repressors are an intracellular read-out of extracellular glucose’s current concentration. By binding these pairs of repressors with appropriate strengths, those *HXT*s with an affinity best matching extracellular glucose are expressed (Fig 3C). High-affinity *HXT*s express only in low glucose because Mig1 and Mig2 prevent their expression in higher concentrations; medium-affinity *HXT*s express only in medium glucose because Mth1 and Std1 prevent their expression in low glucose and Mig1 and Mig2 do so in high glucose; low-affinity *HXT*s express only in high glucose because Mth1 and Std1 prevent their expression in lower concentrations.

At the finer scale of the individual *HXT*s, regulation is more nuanced. Although we believe our methodology would reveal these details with further deletion-strain experiments, the plate-reader data we gathered earlier [31] already suggests that the higher affinity sensor Snf3 predominately inactivates Mth1 and that the lower affinity sensor Rgt2 predominately inactivates Std1, consistent with their strength of interaction [45] and that only Std1 is likely present in higher glucose concentrations because of Mth1’s degradation [44]. Together with our results on Mig1 and Mig2, this absence of Mth1 then suggests that Std1 is the repressor predominately controlling the low-affinity transporters’ expression.

Although at lower levels than the optimal transporters, cells also express Hxts with affinities that match less well the extracellular glucose concentration even in steady conditions (Fig 1B). Perhaps this behaviour is bet-hedging, with cells trading a lower fitness in the current environment for a fast response and so a higher fitness in a possible future one, or Pavlovian-like conditioning [29], with one class of glucose concentration prompting cells to prepare for another. If so, our data suggest that cells anticipate higher glucose concentrations in 0.01% and 0.1% glucose and lower concentrations in 1%.

An implicit underlying assumption of these arguments is that some constraint prevents cells from expressing all the *HXT*s at once, an effective but inefficient mechanism to ensure that the best transporter is always present. One possibility is that the amino acids constituting poorly transporting Hxts are better used elsewhere, for example in ribosomes [46,47]; a second is that space on the plasma membrane is limiting [62].

An atypical *HXT* is *HXT5*, which expresses in the absence of glucose (Fig 1C) and is likely not part of the Snf3-Rgt2 network [7] (Fig G panel B in S1 Text). We suspect that Hxt5 both primes the cells’ response, providing the initial flux of intracellular glucose, and enables cells to skip gears, returning to the introduction’s bicycle analogy. When starving cells sense medium or high levels of glucose, then Snf3 and Rgt2 will act so cells express both the high- and medium-affinity transporters, even though the high-affinity transporters are unnecessary for these glucose concentrations. With sufficient metabolism of the glucose, Mig1 and Mig2 will repress the high-affinity transporter genes, but Hxt5, with its medium affinity, may allow glycolysis to initiate faster and so Mig1 and Mig2 to activate sooner, preventing the levels of the high-affinity transporters ever becoming substantial.

A caveat to our work is that our image analysis pipeline was unable to distinguish Hxt-GFP at the plasma membrane from that being degraded in the vacuole (Fig A panel B in S1 Text), potentially biasing some model parameters. We expect that following Hxt only at the plasma membrane would quantitatively but not qualitatively change the experimental results. Our conclusions because of their dependence on transcriptional control would be unaltered. By focusing on the mother compartment of the cell, a further caveat is that we did not investigate the differential regulation of the *HXT*s between the mother and the bud [53].

Considering the overall network, we suggest that budding yeast has multiple *HXT*s and regulates them by their affinity so that it is competitive through its ability to sequester glucose from others. Comparing the yield, growth rate, and rate of glucose import from 46 yeast species growing in 2% glucose [12], *Saccharomyces cerevisiae* excels only at importing glucose (Fig K in S1 Text). Such selfish strategies are evolutionarily stable in principle [16], and having high numbers of *HXT* genes correlates across yeast species with aerobic fermentation [26], an inefficient but rapid means to generate ATP from glucose [37].

The solution to match *HXT* expression to the glucose concentration may be a convergent one. The *HXT* network generates two pairs of repressors whose concentrations when active change with glucose monotonically and in opposite ways, one increasing and one decreasing. Their nuclear concentration maps to the extracellular glucose concentration, and the *HXT*s express in particular concentration ranges through how their promoters read the two types of repressors. This behaviour mirrors the regulation acting in a different setting, the early development of the *Drosophila* embryo [52]. There the embryo’s task is, broadly, to match expression of segmentation genes to a restricted spatial range [9]. Analogous to the behaviours of Mth1 and Std1 and of Mig1 and Mig2, a component of the solution is to have transcription factors whose concentration changes with space monotonically and in opposite ways, some decreasing with distance from the embryo’s anterior pole, such as *bicoid*, and some increasing, such as *nanos*. Their concentration maps to a spatial location [9], and the different gap genes express in particular spatial ranges through how their promoters read these transcriptional regulators [52]. We expect that such regulatory solutions to match gene expression to the values of an extracellular cue are widespread.

## Materials and methods

### Media and strains

All strains are derived from the *Saccharomyces cerevisiae* BY4741 laboratory strain. We tagged *HXT* genes at their C-terminals with yeast-enhanced GFP and first generated deletions via PCR-based integration and then via a synthetic genetic array to obtain combinations of each of *HXT1-7* in every deletion background (S1 Text). Each strain had one GFP-tagged *HXT*. For microscopy, cells were grown in low-fluorescence synthetic complete medium [49].

### Microscopy

We performed all microscope experiments on a Nikon Eclipse Ti inverted microscope with filter sets for GFP and Cy5, imaging every five minutes. We used Cy5 as a marker for glucose, adding it to the glucose medium. The microscope stage was inside an incubation chamber (Okolabs) held at a constant temperature of 30^°^C. We used a 60X 1.2NA water immersion objective, the Nikon Perfect Focus System (PFS), and an Evolve EMCCD camera (Photometrics). We created five-chamber ALCATRAS devices [11] into which we delivered media using an external mixer (STC) and two syringe pumps (New Era Pump Systems, model NE100).

### Data analysis

We segmented and tracked cells over time from bright-field images using the DISCO algorithm [1]. Glucose concentrations were inferred from the Cy5 signal (S1 Text). We standardised fluorescence measurements using a Mig1-GFP strain and an untagged BY4741 strain placed in two of the five flow chambers. To correct for autofluorescence, we subtracted the fluorescence averaged over all cells in the BY4741 chamber from the fluorescence of each GFP-tagged cell; to correct for day-to-day variations in imaging, we divided the fluorescence of the GFP-tagged cells by the fluorescence averaged over all cells in the Mig1-GFP chamber but only for those time points before we added glucose (S1 Text).

We analysed data from the mother compartments of cells, taking the mean of the fluorescence of all their constituent pixels. The bud’s distribution of Hxts is likely to be different [53].

### Mechanistic modelling

The mathematical model comprises five differential equations and one algebraic equation (S1 Text). We simulated the model in Julia using the OrdinaryDiffEq library with the TRBDF2 solver and fitted using a weighted sum of squares and a black box optimiser. To compare different promoter models, we used the ABC-SMC algorithm [55] run with a customised Adaptive Population Monte Carlo (APMC) algorithm [23] (S1 Text).

### Supporting information

S1 Text Dynamic patterns of gene expression match extracellular signals through push-pull regulation. (PDF)
