# Supplementary material for: Dynamic patterns of gene expression match extracellular signals through push-pull regulation
Source: PLoS Genet. 2025 Nov 19;21(11):e1011943. doi: 10.1371/journal.pgen.1011943 (PMC12668610; doi:10.1371/journal.pgen.1011943)
Supplement: S1 Text — (PDF) [file pgen.1011943.s001.pdf]

Supporting information: Dynamic patterns of gene expression match extracellular signals through push-pull regulation

Luis Fernando Montaño-Gutierrez[1], Marc Sturrock[2], Iseabail L. Farquhar[1], Kevin Correia[1], Vahid Shahrezaei[3], and Peter S. Swain[1]

[1]School of Biological Sciences, University of Edinburgh, United Kingdom
[2]Royal College of Surgeons in Ireland, Dublin, Ireland
[3]Department of Mathematics, Imperial College London, United Kingdom

Contents

1 Materials and Methods **2**
1.1 Media, plasmids, and strains . . . 2
1.1.1 Media . . . 2
1.1.2 Plasmids . . . 2
1.1.3 Strains . . . 2
1.1.4 Performing gene deletions and C-terminal protein tagging . . . 2
1.1.5 Culturing cells . . . 4
1.2 Time-series measurements using microscopy . . . 4
1.2.1 Preparing the cultures . . . 4
1.2.2 Performing the microscopy . . . 4
1.2.3 Media . . . 5
1.2.4 Making microfluidic devices . . . 5

2 Measured affinities of the Hxts **7**

3 Reconstructing the HXT phylogeny **7**

4 Analysis of microscopy data **7**
4.1 Automated segmentation of cells . . . 7
4.2 Estimating the time series of glucose . . . 7
4.3 Obtaining a standardised fluorescence . . . 8
4.4 Steady-state microscopy experiments . . . 10
4.5 Calculating phenotypic distances using mutual information . . . 10

5 Mechanistic modelling **11**
5.1 Transcriptional regulation spikes the levels of Hxt4 . . . 11
5.2 Modelling HXT4 . . . 12
5.2.1 Imposing priors on the parameters controlling regulation . . . 14
5.2.2 Simulating and fitting the model for Hxt4 . . . 15
5.2.3 Optimal values of the parameters and initial conditions . . . 17
5.2.4 Parameter sensitivities . . . 18
5.3 Fitting the other HXTs given HXT4's regulatory model . . . 20
5.4 Caveats to the fitting . . . 22

6 Predicting transcriptional regulation from the promoter sequences **22**

1 Materials and Methods

1.1 Media, plasmids, and strains

1.1.1 Media

Abbreviation	Composition	Use
SC	0.2% Yeast Nitrogen Base (YNB)<br>0.5% ammonium sulfate	Pre-cultures
LFSC	YNB w/o riboflavin, folic acid<br>0.5% ammonium salts	Plate reader
XY Glucose	YEP + 0.1% adenine<br>+ 0.2% tryptophan + 2% glucose	Yeast transformation
LB	Lysogeny broth	*E. coli* transformation
SCM	SC + 0.002% BSA + 2 mg/l cy5	microfluidics

Table A. Growth media used in our study. We use low-fluorescence synthetic complete (SC) medium [1].

1.1.2 Plasmids

Plasmid name	rep. origins,markers (ec) [y]	source
PKT128	ColE1 (AMP)[HIS]	Addgene
pFA6a-KAN	ColE1 (AMP)[KAN]	Addgene
pFA6a-Hph	ColE1 (AMP)[Hph]	Addgene

Table B. Plasmids used in our study.

1.1.3 Strains

All strains (Table C) are derived from BY4741, which we refer to as wild type.

1.1.4 Performing gene deletions and C-terminal protein tagging

To generate HXT-GFP strains in wild-type and different deletion backgrounds, we first made individual tags of HXT genes and deletions via PCR-based integration of constructs [2, 3] and then via a synthetic genetic array to obtain combinations of each of HXT1-7 in every deletion background.

We chose yEGFP as the fluorophore following previous work [4]. For C-terminal tagging, we obtained plasmids pKT128 [1] from Addgene. We amplified the fluorescence marker cassettes by PCR, transformed the PCR product into yeast, and tested positive colonies by PCR and sequencing.

We created deletions also by PCR-based excision of the ORF of interest. Deletion::Hph strains were generated by amplifying the hph cassette from pYM40 [5] by PCR with overhangs to the ORF of interest and then transforming the query strain for the synthetic genetic array [6]. We verified colonies resistant to the antibiotic by both PCR and sequencing from both edges of the integration site.

SGA for double tags and tagged deletions: We generated a synthetic genetic array (SGA) library of GFP-tagged and deletion strains following an established procedure [6], involving crossing tagged strains in the BY background with the deletion made in the SGA query strain. We planned our markers to maximise compatibility between HIS markers with a TEF promoter and terminator (plasmid pKT128) and a hph marker driven by an ADH promoter and terminator (plasmid pYM40).

Strain ID	in–text description	Genotype or background
SL077/SL229	BY4741 (WT)	MATa his3Δ1 leu2Δ0 ura3Δ0 met15Δ0
SL567	SGA query strain Y6547	Matα can1Δ::pMFA1-LEU2 lyp1Δ ura3Δ0 leu2Δ0 his3Δ1 met15Δ0
SL621	rgt2Δ	SL567 rgt2::Hph
SL612	std1Δ	SL567 std1::Hph
SL618	mth1Δ	SL567 mth1::Hph
SL668	mig1Δ	SL567 mig1::Hph
SL620	mig2Δ	SL567 mig2::Hph
SL614	snf3Δ	SL567 snf3::Hph
SL498	Hxt1-GFP	SL229 HXT1-yEGFP::HIS
SL480	Hxt2-GFP	SL229 HXT2-yEGFP::HIS
SL485	Hxt3-GFP	SL229 HXT3-yEGFP::HIS
SL409	Hxt4-GFP	SL229 HXT4-yEGFP::HIS
SL487	Hxt5-GFP	SL229 HXT5-yEGFP::HIS
SL488	Hxt6-GFP	SL229 HXT6-yEGFP::HIS
SL566	Hxt7-GFP	SL229 HXT7-yEGFP::HIS
SL747	Hxt4-GFP mig1Δ	SL409 x SL668
SL748	Hxt4-GFP rgt2Δ	SL409 x SL621
SL749	Hxt4-GFP std1Δ	SL409 x SL612
SL796	Hxt4-GFP mth1Δ	SL409 x SL618
SL798	Hxt4-GFP snf3Δ	SL409 x SL614
SL957	Hxt1-GFP rgt2Δ	SL498 x SL621
SL959	Hxt1-GFP std1Δ	SL498 x SL612
SL956	Hxt1-GFP mth1Δ	SL498 x SL618
SL798	Hxt1-GFP snf3Δ	SL498 x SL614
SL961	Hxt2-GFP rgt2Δ	SL480 x SL621
SL963	Hxt2-GFP std1Δ	SL480 x SL612
SL960	Hxt2-GFP mth1Δ	SL480 x SL618
SL962	Hxt2-GFP snf3Δ	SL480 x SL614
SL977	Hxt2-GFP rgt2Δ	SL485 x SL621
SL979	Hxt2-GFP std1Δ	SL485 x SL612
SL976	Hxt2-GFP mth1Δ	SL485 x SL618
SL978	Hxt2-GFP snf3Δ	SL485 x SL614
SL965	Hxt5-GFP rgt2Δ	SL487 x SL621
SL967	Hxt5-GFP std1Δ	SL487 x SL612
SL964	Hxt5-GFP mth1Δ	SL487 x SL618
SL966	Hxt5-GFP snf3Δ	SL487 x SL614
SL969	Hxt6-GFP rgt2Δ	SL488 x SL621
SL971	Hxt6-GFP std1Δ	SL488 x SL612
SL968	Hxt6-GFP mth1Δ	SL488 x SL618
SL970	Hxt6-GFP snf3Δ	SL488 x SL614
SL973	Hxt7-GFP rgt2Δ	SL566 x SL621
SL975	Hxt7-GFP std1Δ	SL566 x SL612
SL972	Hxt7-GFP mth1Δ	SL566 x SL618
SL974	Hxt7-GFP snf3Δ	SL566 x SL614
SL719	pHXT4-GFP	SL229 pHXT4-yEGFP@ura3

Table C. Strains used in our study.

Genomic sequences: We obtained promoter and primer sequences from the *Saccharomyces* reference genome version R64-2-1 for strain S288C through the Saccharomyces Genome Database [7].

Assembly using MoClo: To generate a construct with the HXT4 promoter driving a GFP, we used the MoClo system [8]. We designed MoClo compatible synthetic versions of the HXT promoters and for yEGFP. We extracted the 1,000 bp upstream of the first codon of the HXT4 ORF. We then adapted and modified the sequence for DNA synthesis and MoClo assembly in three steps: 1) we removed BsmB1, Bsa1, and Not1 sites; 2) we shortened the length of single nucleotide repeats using the standards of Gen9 (now Gingko Bioworks); 3) we added MoClo assembly overhangs. The overhangs consist of an outer BsmBI site and an inner BsaI site followed by part-specific four-letter overhangs. We assembled the promoter, GFP ORF, and terminator parts into the pYTK001 MoClo entry vector using BsmBI assembly [8].

1.1.5 Culturing cells

We typically prepared pre-cultures by inoculating single yeast colonies in SC medium complemented with 2% of the carbon source for 24 hours. The incubation medium was always SC to minimise effects from auxotrophy [9]. We agitated 5 ml overnight cultures in test tubes at 260 rpm. After 24 hours, this treatment yielded an OD_{595} greater than 1 for 2% galactose and typically an OD of 0.3-0.4 for 2% pyruvate. For the experiments of Fig 1B, we inoculated 100 μl of the overnight culture into 5 ml of SC with 2% pyruvate as a carbon source, to avoid any induction of the HXTs by hexoses, and grew cells for around 8 hours, keeping the OD below 0.1. For the experiments of Fig 1C and Fig 2, we inoculated approximately 30 μl of cells into a 5 ml of SC containing 2% galactose and grew cells for 6-8 hours, again keeping the OD below 0.1. We then loaded cells into a microfluidic device containing SC medium and flowed SC medium for three hours before adding glucose.

1.2 Time-series measurements using microscopy

1.2.1 Preparing the cultures

For all microscopy experiments unless otherwise specified, we started from a 24 hour pre-culture in 2% galactose. We diluted this pre-culture into fresh SC medium with 2% galactose and incubated for 7-9 hours to reach an OD between ~0.1 and ~0.2. We directly inoculated the culture into a microfluidic device loaded with sugarless SC medium.

1.2.2 Performing the microscopy

We performed all microscope experiments on a Nikon Eclipse Ti inverted microscope with filter sets for GFP and Cy5 imaging. We used Cy5 as a marker for glucose, adding Cy5 to the glucose medium. The microscope stage was inside an incubation chamber (Okolabs) held at a constant temperature of 30°C. We used a 60X 1.2NA water immersion objective, the Nikon Perfect Focus System (PFS), and an Evolve EMCCD camera (Photometrics).

In all the experiments unless otherwise specified, we imaged every five minutes. Each imaging cycle included one Cy5 acquisition and five bright-field and GFP stacks per position. We kept GFP imaging at a low intensity (1 volt and 30 milliseconds) to reduce phototoxicity. We controlled image acquisition using bespoke code in MATLAB (Mathworks) and with MicroManager [10].

1.2.3 Media

For microfluidics experiments, we topped 39.76 ml of the medium with 240 μl of a Cy5/BSA or ddH_2O/BSA solution. We added BSA (bovine serum albumin) to reduce clogging of cells because BSA blocks reactive groups in the polydimethylsiloxane (PDMS) polymer constituting the microfluidic device [11].

1.2.4 Making microfluidic devices

We created ALCATRAS five chamber devices [11] through standard soft lithography [12]. We filled a rigid master mould containing device negatives with a degassed mixture of PDMS and silicone curing agent at a ratio of 10:1 and then cast at 65°C for at least four hours. We cut out the positive device cast and punched media inlet holes with a 1 mm biopsy punch. We bonded the device to a microscope coverslip with 30 seconds of plasma treatment and primed it by filling with the medium used at the beginning of the experiment, typically with no sugar.

We used two syringe pumps to deliver media into the device (New Era Pump Systems, model NE100). The total flow rate was 4 $\mu\ell$/min throughout the imaging period. Mixing from both media pumps was through an external mixer (STC). To minimise exposure of cells to trace amounts of glucose, we flowed the medium containing 0% glucose for three or more hours before loading the cells. A dynamic mixing programme adjusted the pumps in real time through custom MATLAB scripts and the pumps' software. We manually injected cells into the device with a syringe.

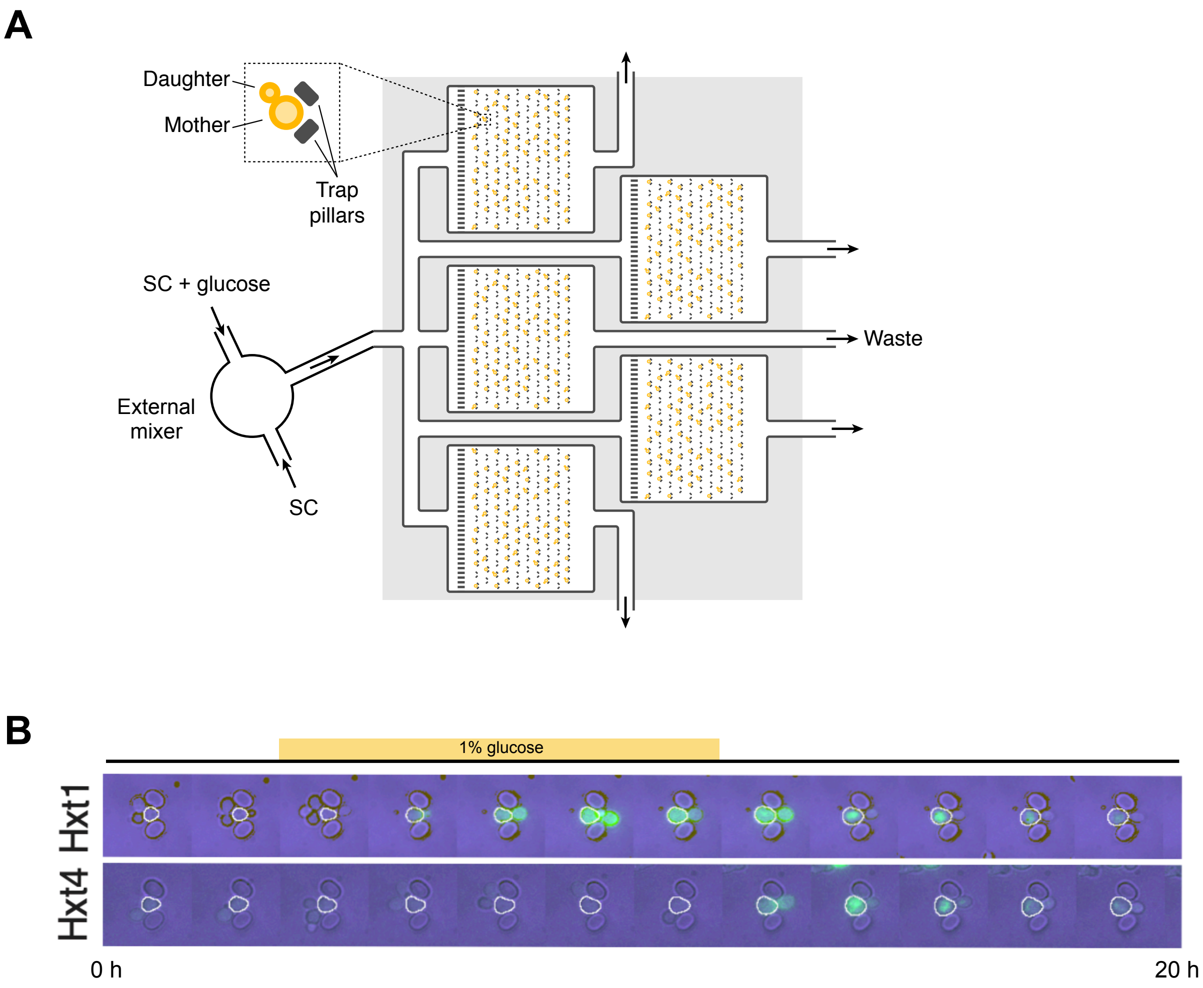

Fig A. We use microfluidics to follow Hxt-GFP levels in hundreds of cells in controlled but dynamic glucose environments. **A** A schematic of the five-chamber microfluidic ALCATRAS device we used, based on that from Granados *et al.* [13]. In a typical experiment, we monitored three strains each with a different HXT tagged with GFP in three chambers and used the other two chambers for controls for standardisation: a MIG1-GFP and an untagged BY4741 strain (Sec. 4.3). All chambers received the same medium, and we used an external mixer to switch between SC medium without glucose and SC medium with. The inset shows a schematic of an ALCATRAS trap, where a mother cell sits stably between two pillars of transparent PDMS polymer [11]. There are hundreds of traps per chamber. **B** Time-lapse microscopy images of cells in a single trap of a ALCATRAS microfluidic device show the distinct behaviours of different GFP-tagged Hxts. Data are from the experiment in Fig 1C where glucose is present for eight hours, and we have superimposed bright-field and fluorescence images, using the maximum projection over five Z slices for the fluorescence image. Once we removed glucose, cells internalised and degraded these particular Hxts. We outline in white the central cell, the one from which we analyse data taking the mean fluorescence over all its constituent pixels.

2 Measured affinities of the Hxts

Transporter	Methodology				Average
	counter-transport [14]	initial uptake [14]	5 mM [15]	100 mM [15]	
Hxt1	107	129	90	110	109
Hxt2	2.9	4.6	1.5	10	4.75
Hxt3	28.6	34.2	55	55	43.2
Hxt4	6.2	6.2	9.3	9.4	7.8
Hxt6	0.9	1.4	2.5	2.5	1.8
Hxt7	1.3	1.9	1.1	2.1	1.6

Table D. Reported values for the K_M, the inverse of the affinity, for all Hxts known to be expressed in glucose. A well-known exception, Hxt5, has a K_M of 10 mM [16].

3 Reconstructing the HXT phylogeny

We constructed the phylogeny of fungal hexose transporter homologues with the ontology terms PTHR23500 and PTHR23503:SF8 (PTHR14.1) [17] using MAFFT [18] and IQ-Tree [19] with their default parameters. We included *Bacillus subtilis* homologues as an out group. This analysis resulted in two clades of the dominant hexose transporter family in *S. cerevisiae*: the HXT clade and a clade with RGT2 and SNF3. We created additional reconstructions for each clade to better understand their evolution.

We reconstructed the phylogeny of the HXT clade (PTHR23500:SF131) with homologues in *S. cerevisiae* S288c, *Candida albicans* SC5314, and from yeasts that did not undergo the whole genome duplication: *Phaffomycetaceae*, *Saccharomycodaceae*, *Lachancea*, *Eremothecium*, *Kluyveromyces*, *Torulaspora*, *Zygosaccharomyces*, and *Zygotorulaspora*. We excluded yeasts that had undergone the whole genome duplication as they have multiple HXT homologues that are irrelevant to reconstructing the evolution of HXT1-7. Including all the sequenced species in these lineages rather than a single species reduces selection bias. For example, say HXT1 is present in *S. cerevisiae* and *Zygosaccharomyces bailii*, but not in *Zygosaccharomyces rouxii*, then comparative genomics with *S. cerevisiae* and *Z. rouxii* would falsely indicate that HXT1 is exclusive to *S. cerevisiae*, but not comparative genomics with *S. cerevisiae* and the *Zygosaccharomyces* lineage.

From analysing the HXT phylogeny and synteny, we concluded that HXT1 and HXT3 are ohnologues, rather than the current annotation of HXT1 and HXT6. HXT6 and HXT7 are paralogous. HXT5 does not have an ohnologue: it was lost in *S. cerevisiae*'s whole-genome-duplicated ancestor.

4 Analysis of microscopy data

4.1 Automated segmentation of cells

We segmented and tracked cell areas from the bright-field images using the DISCO algorithm [20].

4.2 Estimating the time series of glucose

We obtained the glucose signal by adding a Cy5 dye in all glucose media. We measured the Cy5 signal by averaging those pixels in the image not assigned to cells. Unfortunately Cy5 appeared to bind to the PDMS polymer constituting the microfluidic devices and generated a residual signal even when we removed glucose.

We therefore processed the Cy5 signal to correct for this binding to PDMS. For the step experiments (Fig 1B) and the 'hat' experiments — ones with increasing, constant, and then decreasing glucose (Fig 1C), we used two different procedures.

For the steps, the corrected signal is binary, corresponding to either no or maximal glucose. We identified when the step in glucose occurs by the time point where the Cy5 signal had its maximal time derivative, using a Matern Gaussian process to infer the derivatives [21].

For the hats, we generated a corrected signal that is locally symmetric around the mid-point of the Cy5 signal's plateau region. We took the maximal values of the Cy5 signal's absolute time derivative to find the time points where glucose increased and decreased. During the glucose plateau, we let the corrected signal have the maximal value of the Cy5 signal; both before glucose increased and after it decreased, we let the corrected signal have the minimal value of the Cy5 signal. By reflecting around the midpoint of the glucose plateau, we forced the signal to be locally symmetric. We identified the midpoint as the time point halfway between the two time points where the Cy5 signal had its maximal absolute time derivative. Next, we smoothed the signal with a first-order spline and optimised the duration of the signal's plateau by a best match with the measured Cy5 signal in the regions where Cy5 was increasing or decreasing. We used least squares for the optimisation.

Finally, we estimated the glucose signal by scaling the Cy5 signal to have a minimal value of zero and a maximal value of the maximal glucose concentration used in an experiment.

4.3 Obtaining a standardised fluorescence

To measure fluorescence robustly across multiple experiments, we ran experiments with two control strains (Fig B): the untagged wild type BY4741 and MIG1-GFP (strain SL518). We chose MIG1-GFP because we noted that the levels of Mig1-GFP were approximately constant in the sugarless medium prior to glucose exposure. To standardise fluorescence, we subtracted the mean of the wild type at every position for all cells, correcting for autofluorescence. Then we divided the fluorescence of all cells by the mean level of all Mig1-GFP time series over the first 2–3 hours before we added glucose, approximately 30 time points. The fluorescence is therefore measured in Mig1 units.

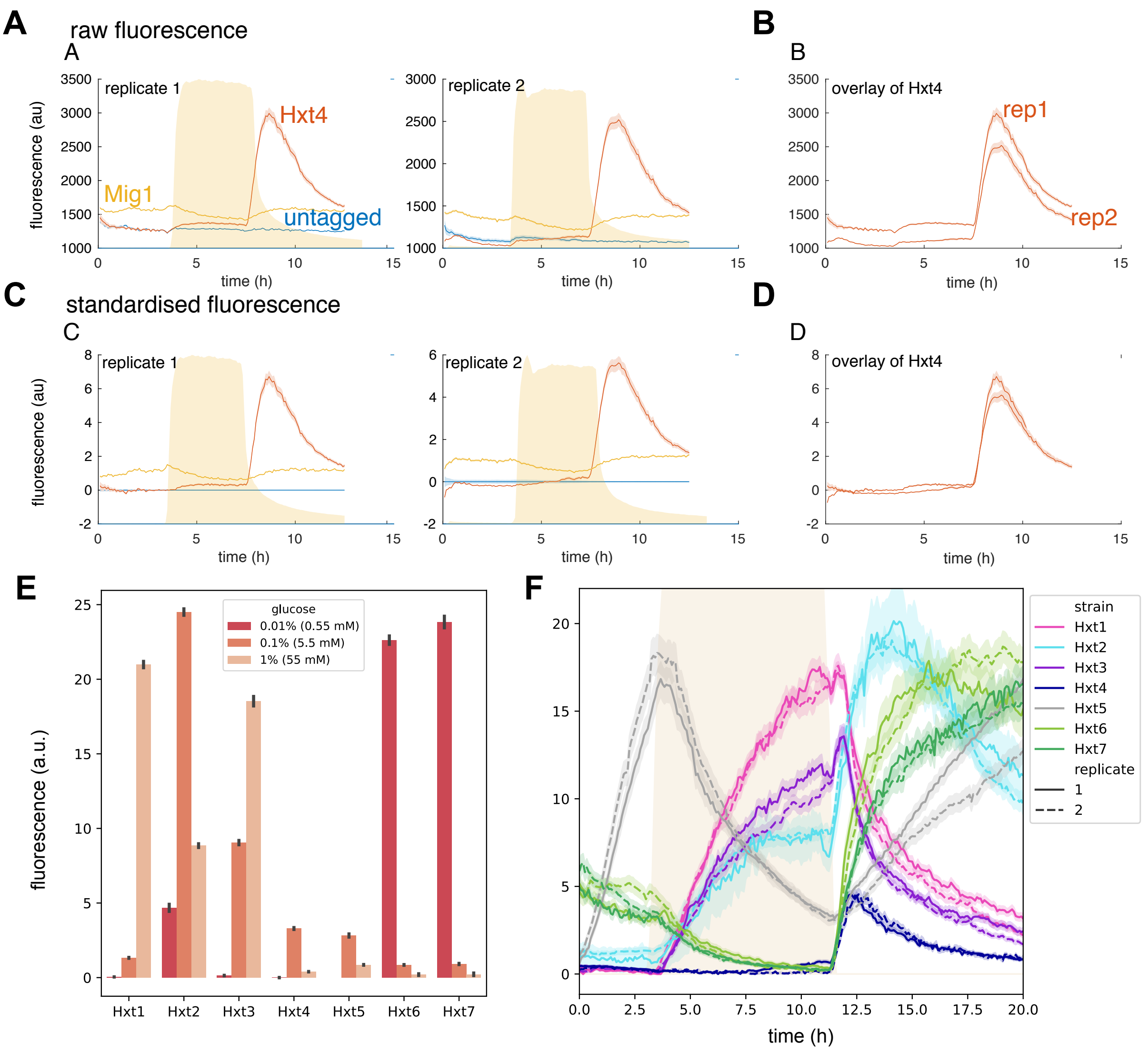

Fig B. Normalising by both the wild-type fluorescence and the fluorescence of Mig1-GFP corrected for experiment-to-experiment variation. For every experiment, we used an untagged wild-type strain to estimate autofluorescence and subtracted its mean over all cells from the mean fluorescence of a HXT-GFP strain. Additionally we included a MIG1-GFP strain to correct any day-to-day variation in the microscopy. We divided the mean fluorescence of the strain with HXT-GFP by the mean fluorescence of the MIG1-GFP strain averaged over the first three hours of the experiment. Fluorescence is then measured in units of the mean Mig1 fluorescence. **A** The data from two replicate experiments. Hxt4-GFP is in red; Mig1-GFP is in orange; and the fluorescence from the untagged wild-type strain is in blue. We show the Cy5 signal with shading. **B** An overlay of Hxt4-GFP from the two experiments. **C** The data once standardised. **D** The behaviour of Hxt4-GFP becomes the same for both experiments. **E** The data of Fig 1B re-plotted to show the corresponding errors. **F** Both replicates for the data in Fig 1C.

In experiments where the MIG1-GFP strain was absent, we first scaled so that the minimal value of the Hxt4-GFP fluorescence is zero and its maximal value is one. Second, to place the data on a Mig1-GFP scale, we multiplied by a proportionality constant — the fluorescence of Hxt4-GFP at its peak divided by the average initial fluorescence of Mig1-GFP using the 1% glucose experiments that had both the MIG1-GFP and HXT4-GFP strains.

Any negative values near zero, arising from measurement noise, were set to zero for model

fitting.

4.4 Steady-state microscopy experiments

After three hours growing in SC medium with 2% galactose, we switched cells in an ALCATRAS microfluidic device to SC medium with either 0.01%, 0.1%, or 1% glucose and imaged for at least another 10 hours.

To estimate the steady-state level of each Hxt, we used a Gaussian process with a neural network kernel to estimate the mean and time derivative of the fluorescence [21] from all the biological replicates of a strain ($2 \leq n \leq 5$). We consider steady state to be all time points after t =14 h that have an estimated time-derivative with an absolute value less than 0.1 fluorescence units per hour. We then defined the steady-state fluorescence as the median over these time points of the mean of the Gaussian process and its error as the median over the same time points of the Gaussian process's standard deviation.

4.5 Calculating phenotypic distances using mutual information

Using mutual information to cluster the single-cell time series [13], we found that the clusters match those anticipated from the mean behaviour of Fig 1C and, excepting Hxt2, from phylogeny (Fig 1A).

As a metric of the phenotypic distance between single cells, we calculated the mutual information between groups of single-cell fluorescence time series using an established algorithm [13]. We defined a cell's identity as the particular HXT gene tagged with GFP. We then ask how accurately we can determine a cell's identity by only observing its fluorescence time series. A pair of strains whose single-cell time series are statistically indistinguishable will produce a mutual information of zero; strains with distinct time series will yield a mutual information of one. We used the R library written by Julian Pietsch [13] with the default settings [13].

A caveat is the smaller numbers of time series we used compared to the original work. As a consequence, the mutual information of a strain with itself is only close to and not zero. We therefore called the mutual information a pseudo-distance rather than a distance.

Generation of phenotypic pseudo-distance tree: To generate a pairwise comparison, we computed the mutual information for every pair of HXTs using the experimental replicate with the largest number of cells: To make the tree shown in Fig C, we considered the diagonal of

	Hxt1	Hxt2	Hxt3	Hxt4	Hxt5	Hxt6	Hxt7
Hxt1	0.0176	0.4896	0.0867	0.4582	0.4520	0.7353	0.5285
Hxt2		0.0133	0.4852	0.6300	0.4665	0.6444	0.5654
Hxt3			0.0234	0.4039	0.4896	0.7256	0.5245
Hxt4				0.0384	0.6352	0.7674	0.5895
Hxt5					0.0110	0.3426	0.2369
Hxt6						0.0230	0.0536
Hxt7							0.0115

the matrix to be negligibly small and set all diagonal terms to zero. By plotting the output of R's `hclust` function with the pseudo-distance matrix as an input, we produced a dendrogram. Fig C preserves the topology of the dendrogram, rooted at HXT5.

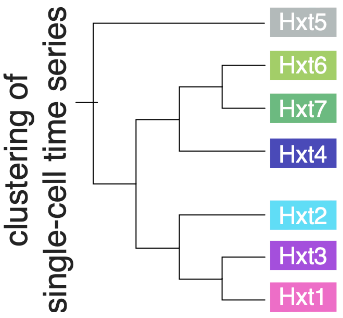

Fig C. Single cells behave similarly to their mean response. Clustering the single-cell time series from Fig 1C shows the relationships between the mean behaviours of the different Hxts holds too at the single-cell level and mostly reproduces the phylogenetic relationships of Fig 1A.

5 Mechanistic modelling

5.1 Transcriptional regulation spikes the levels of Hxt4

We first determined that transcriptional regulation drives the behaviour of Hxt4-GFP (Fig D).

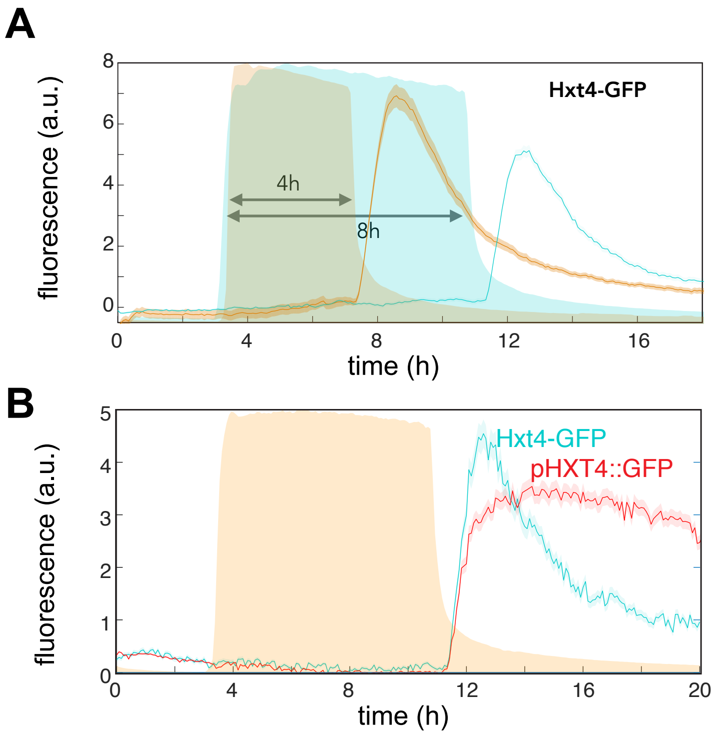

Fig D. Spiking in Hxt4 is insensitive to the glucose input's duration and is predominately generated by transcriptional regulation. A Our usual input involved a switch into glucose for approximately eight hours. For a switch into 1% glucose, levels of Hxt4 spiked only when glucose fell. Decreasing the period of constant glucose to four hours generated a similar spike. The behaviour is therefore not generated by a delay, for example by glucose diluting or degrading a repressive factor. We use shading to show glucose. **B** GFP driven by the promoter of HXT4 exhibited a similar spike in levels and only in decreasing glucose. Post-translational regulation, likely active degradation, of Hxt4 changed the temporal shape of this response, but was not required to generate the spike. The maximal concentration of glucose (shading) was 1%.

5.2 Modelling HXT4

Intracellular glucose: Changes in extracellular glucose induce changes in intracellular glucose, but their relationship will alter as the levels of the Hxts change.

To include such effects, we assumed that the changes in extracellular glucose applied in the microfluidic device are immediate, with a rapid rise in extracellular glucose concentration in all experiments (Fig 1B & C) and a rapid fall in some (Fig 1C). We defined intracellular glucose to be minimal when extracellular glucose was zero and to be an increasing logistic function after a rise in extracellular glucose at $t = t_{\mathrm{up}}$:

$$g_i = \frac{g_{\max}}{1 + \frac{g_{\max} - g_{\min}}{g_{\min}} \mathrm{e}^{-r(t - t_{\mathrm{up}})}}. \tag{1}$$

We set $g_{\max}$ to be the maximal extracellular glucose concentration in a particular experiment and $g_{\min}$ to be negligible, arbitrarily 10^{-7} %. In a fall in extracellular glucose, we assume any intracellular glucose is rapidly consumed, becoming minimal again in one time point.

SNF1: Metabolised glucose inhibits the kinase complex SNF1 [22]. We assumed this inhibition occurs through allosteric regulation, which gives a simple mathematical expression if the levels of SNF1 do not change [23]. We include that Std1 binds SNF1 [24, 25] and enhances SNF1's activity [24–26] independently of glucose.

Writing the fixed amount of SNF1 as $\mathrm{SNF1}_{\mathrm{tot}}$, g_i as intracellular glucose, K_{SNF1Std1} as the dissociation constant of Std1 binding, and K_{SNF1} as the dissociation constant of glucose binding, then active SNF1 satisfies:

$$\mathrm{SNF1} = \frac{\mathrm{SNF1}_{\mathrm{tot}} \left(1 + \frac{\mathrm{Std1}}{K_{\mathrm{SNF1Std1}}}\right)^{n_{\mathrm{SNF1}}}}{\left(1 + \frac{\mathrm{Std1}}{K_{\mathrm{SNF1Std1}}}\right)^{n_{\mathrm{SNF1}}} + L\left(1 + \frac{g_i}{K_{\mathrm{SNF1}}}\right)^{m_{\mathrm{SNF1}}}}. \tag{2}$$

The m and n parameters determine the cooperativity of binding, and L determines the fraction of active SNF1 when both g_i and Std1 are zero.

Mig1: When glucose concentrations are low, active SNF1 phosphorylates Mig1, causing its exit from the nucleus [27] and inhibiting its repression of the HXTs. With i_{Mig1} as the rate of entry to the nucleus and e_{Mig1} as the rate of exit, we model nuclear Mig1 using:

$$\frac{d\mathrm{Mig1}}{dt} = i_{\mathrm{Mig1}}(\mathrm{Mig1}_{\mathrm{tot}} - \mathrm{Mig1}) - \frac{e_{\mathrm{Mig1}} \cdot \mathrm{SNF1} \cdot \mathrm{Mig1}}{K_{\mathrm{Mig1SNF1}} + \mathrm{Mig1}}. \tag{3}$$

We include SNF1's effects by a Hill function with a Hill number of 1.

Mig2: The MIG2 gene is repressed by Rgt1 [28], expressing in the presence of glucose when Mth1 and Std1 become inactivated. For simplicity, we do not explicitly include translation, and our synthesis term directly produces proteins, assuming levels of mRNA quickly attain steady state. We use

$$\frac{d\mathrm{Mig2}}{dt} = \frac{s_{\mathrm{Mig2}}}{1 + \left(\frac{\mathrm{Mth1}}{K_{\mathrm{MIG2Mth1}}}\right)^{n_{\mathrm{MIG2Mth1}}} + \left(\frac{\mathrm{Std1}}{K_{\mathrm{MIG2Std1}}}\right)^{n_{\mathrm{MIG2Std1}}}} - \left[d_{\mathrm{Mig2}} + \frac{d_{\mathrm{Mig2g}}}{1 + \left(\frac{g_i}{K_{\mathrm{Mig2g}}}\right)^{m_{\mathrm{Mig2}}}}\right] \mathrm{Mig2} \tag{4}$$

where we model the genetic regulation by Hill-like functions.

We include both a constant rate of degradation, determined by d_{Mig2}, and glucose-inhibited degradation, determined by d_{Mig2g}, so that intracellular glucose, g_i, promotes higher levels of Mig2. Without this glucose-regulated degradation, our fits to the data were worse.

Mth1: Extracellular glucose, g_e, enhances the co-repressor Mth1's rate of degradation through the two sensors Snf3 and Rgt2 [29]. Mig1 and Mig2 repress MTH1 [28]. We use Hill functions for the regulation:

$$\begin{aligned}\frac{d\text{Mth1}}{dt} =& \frac{s_{\text{Mth1}}}{1 + \left(\frac{\text{Mig1}}{K_{\text{MTH1Mig1}}}\right)^{n_{\text{MTH1Mig1}}} + \left(\frac{\text{Mig2}}{K_{\text{MTH1Mig2}}}\right)^{n_{\text{MTH1Mig2}}}} \\ & - \left[d_{\text{Mth1}} + \frac{d_{\text{Mth1Snf3}} \cdot g_e^{m_{\text{Mth1Snf3}}}}{K_{\text{Snf3}}^{m_{\text{Mth1Snf3}}} + g_e^{m_{\text{Mth1Snf3}}}} + \frac{d_{\text{Mth1Rgt2}} \cdot g_e^{m_{\text{Mth1Rgt2}}}}{K_{\text{Rgt2}}^{m_{\text{Mth1Rgt2}}} + g_e^{m_{\text{Mth1Rgt2}}}}\right] \text{Mth1}\end{aligned} \tag{5}$$

Std1: We model extracellular glucose, via Snf3 and Rgt2 [28, 30], as inactivating Std1 by causing Std1 to leave the nucleus and form puncta near the vacuole [26]. Writing i_{Std1} for the rate of entry to the nucleus and e_{Std1} for the rate of exit:

$$\frac{d\text{Std1}}{dt} = i_{\text{Std1}}(\text{Std1}_{\text{tot}} - \text{Std1}) - \left[\frac{e_{\text{Std1Snf3}} \cdot g_e^{m_{\text{Std1Snf3}}}}{K_{\text{Snf3}}^{m_{\text{Std1Snf3}}} + g_e^{m_{\text{Std1Snf3}}}} + \frac{e_{\text{Std1Rgt2}} \cdot g_e^{m_{\text{Std1Rgt2}}}}{K_{\text{Rgt2}}^{m_{\text{Std1Rgt2}}} + g_e^{m_{\text{Std1Rgt2}}}}\right] \text{Std1} \tag{6}$$

We fix the total level of Std1 to be Std1_{tot} [31].

HXT4: Mth1, Std1, Mig1, and Mig2 all repress HXT4 [28], which we model using a Hill function-like term. We let glucose promote levels of Hxt4 by inhibiting Hxt4's degradation [32]. Let s_{Hxt4} be the synthesis rate. All K parameters are dissociation constants of binding to the HXT4 promoter and all n parameters quantify the cooperativity of binding. The two d parameters determine Hxt4's rate of degradation, where g_i is intracellular glucose and we again use a Hill function to model glucose-inhibited degradation:

$$\begin{aligned}\frac{d\text{Hxt4}}{dt} =& s_{\text{Hxt4}} \Big/ \left[1 + \left(\frac{\text{Mig1}}{K_{\text{HXT4Mig1}}}\right)^{n_{\text{HXT4Mig1}}} + \left(\frac{\text{Mig2}}{K_{\text{HXT4Mig2}}}\right)^{n_{\text{HXT4Mig2}}}\right. \\ & \left. + \left(\frac{\text{Mth1}}{K_{\text{HXT4Mth1}}}\right)^{n_{\text{HXT4Mth1}}} + \left(\frac{\text{Std1}}{K_{\text{HXT4Std1}}}\right)^{n_{\text{HXT4Std1}}}\right] \\ & - \left[d_{\text{Hxt4}} + \frac{d_{\text{Hxt4g}}}{1 + \left(\frac{g_i}{K_{\text{Hxt4g}}}\right)^{m_{\text{Hxt4}}}}\right] \text{Hxt4}.\end{aligned} \tag{7}$$

We use the same equation with different values of the parameters to model the regulation of the other HXTs when generating Fig 3.

Our model in total is then:

$$
\begin{aligned}
\mathrm{SNF1} =& \frac{\mathrm{SNF1_{tot}}\left(1+\frac{\mathrm{Std1}}{K_{\mathrm{SNF1Std1}}}\right)^{n_{\mathrm{SNF1}}}}{\left(1+\frac{\mathrm{Std1}}{K_{\mathrm{SNF1Std1}}}\right)^{n_{\mathrm{SNF1}}} + L\left(1+\frac{g_i}{K_{\mathrm{SNF1}}}\right)^{m_{\mathrm{SNF1}}}} \\
\frac{d\mathrm{Hxt4}}{dt} =& s_{\mathrm{Hxt4}} \Bigg/ \left[1+\left(\frac{\mathrm{Mig1}}{K_{\mathrm{HXT4Mig1}}}\right)^{n_{\mathrm{HXT4Mig1}}} + \left(\frac{\mathrm{Mig2}}{K_{\mathrm{HXT4Mig2}}}\right)^{n_{\mathrm{HXT4Mig2}}} \right. \\
& \left. + \left(\frac{\mathrm{Mth1}}{K_{\mathrm{HXT4Mth1}}}\right)^{n_{\mathrm{HXT4Mth1}}} + \left(\frac{\mathrm{Std1}}{K_{\mathrm{HXT4Std1}}}\right)^{n_{\mathrm{HXT4Std1}}}\right] \\
& - \left[d_{\mathrm{Hxt4}} + \frac{d_{\mathrm{Hxt4g}}}{1+\left(\frac{g_i}{K_{\mathrm{Hxt4g}}}\right)^{m_{\mathrm{Hxt4}}}}\right] \\
\frac{d\mathrm{Mig1}}{dt} =& i_{\mathrm{Mig1}}(1-\mathrm{Mig1}) - \frac{e_{\mathrm{Mig1}}\cdot\mathrm{SNF1}\cdot\mathrm{Mig1}}{K_{\mathrm{Mig1SNF1}}+\mathrm{Mig1}} \\
\frac{d\mathrm{Mig2}}{dt} =& \frac{s_{\mathrm{Mig2}}}{1+\left(\frac{\mathrm{Mth1}}{K_{\mathrm{MIG2Mth1}}}\right)^{n_{\mathrm{MIG2Mth1}}} + \left(\frac{\mathrm{Std1}}{K_{\mathrm{MIG2Std1}}}\right)^{n_{\mathrm{MIG2Std1}}}} \\
& - \left[d_{\mathrm{Mig2}} + \frac{d_{\mathrm{Mig2g}}}{1+\left(\frac{g_i}{K_{\mathrm{Mig2g}}}\right)^{m_{\mathrm{Mig2}}}}\right]\mathrm{Mig2} \\
\frac{d\mathrm{Mth1}}{dt} =& \frac{s_{\mathrm{Mth1}}}{1+\left(\frac{\mathrm{Mig1}}{K_{\mathrm{MTH1Mig1}}}\right)^{n_{\mathrm{MTH1Mig1}}} + \left(\frac{\mathrm{Mig2}}{K_{\mathrm{MTH1Mig2}}}\right)^{n_{\mathrm{MTH1Mig2}}}} \\
& - \left[d_{\mathrm{Mth1}} + \frac{d_{\mathrm{Mth1Snf3}}\cdot g_e^{m_{\mathrm{Mth1Snf3}}}}{K_{\mathrm{Snf3}}^{m_{\mathrm{Mth1Snf3}}}+g_e^{m_{\mathrm{Mth1Snf3}}}} + \frac{d_{\mathrm{Mth1Rgt2}}\cdot g_e^{m_{\mathrm{Mth1Rgt2}}}}{K_{\mathrm{Rgt2}}^{m_{\mathrm{Mth1Rgt2}}}+g_e^{m_{\mathrm{Mth1Rgt2}}}}\right]\mathrm{Mth1} \\
\frac{d\mathrm{Std1}}{dt} =& i_{\mathrm{Std1}}(\mathrm{Std1_{tot}}-\mathrm{Std1}) - \left[\frac{e_{\mathrm{Std1Rgt2}}\cdot g_e^{m_{\mathrm{Std1Rgt2}}}}{K_{\mathrm{Rgt2}}^{m_{\mathrm{Std1Rgt2}}}+g_e^{m_{\mathrm{Std1Rgt2}}}} + \frac{e_{\mathrm{Std1Snf3}}\cdot g_e^{m_{\mathrm{Std1Snf3}}}}{K_{\mathrm{Snf3}}^{m_{\mathrm{Std1Snf3}}}+g_e^{m_{\mathrm{Std1Snf3}}}}\right]\mathrm{Std1}
\end{aligned}
$$

5.2.1 Imposing priors on the parameters controlling regulation

Based on the literature and our data, we impose multiple *a priori* constraints on the parameters:

1. We set $\mathrm{Mig1_{tot}} = 1$ because we normalise our data by the mean Mig1-GFP fluorescence. We also set $\mathrm{SNF1_{tot}} = 1$ because it is mathematically redundant in our model with e_{Mig1} (Eqs. 2 & 3). The value of e_{Mig1} that we fit is therefore an effective one, being the product of its true value and the true value of $\mathrm{SNF1_{tot}}$.

2. The reported median number of Mth1 molecules is 3600, of Std1 molecules is 700, and of Mig1 molecules is 2000 [33]. Considering the number of Mig1 molecules, we imposed an upper bound on the maximal number of Mth1 molecules, $s_{\mathrm{Mth1}}/d_{\mathrm{Mth1}} < 10$, and on Std1 molecules, $\mathrm{Std1_{tot}} < 1$.

3. Snf3 has a lower affinity for glucose than Rgt2 [34], and so we fit $\theta_{\mathrm{Rgt2Snf3}}$ rather than K_{Rgt2} where
$$K_{\mathrm{Rgt2}} = K_{\mathrm{Snf3}} + \theta_{\mathrm{Rgt2Snf3}} \tag{9}$$
with $\theta_{\mathrm{Rgt2Snf3}}$ positive so that $K_{\mathrm{Rgt2}} > K_{\mathrm{Snf3}}$.

4. We assume that Rgt2 affects Std1 more than Mth1, consistent with our earlier measurements [35], because Rgt2 is active in high glucose when the cell has degraded most Mth1

and repressed MTH1. We fit θ_{Rgt2} rather than e_{Std1Rgt2} where

$$e_{\text{Std1Rgt2}} = d_{\text{Mth1Rgt2}} + \theta_{\text{Rgt2}} \tag{10}$$

with θ_{Rgt2} positive.

5. We assume that Snf3 affects Mth1 more than Std1 [35], and so we fit θ_{Snf3} rather than d_{Mth1Snf3} where

$$d_{\text{Mth1Snf3}} = e_{\text{Std1Snf3}} + \theta_{\text{Snf3}} \tag{11}$$

with θ_{Snf3} positive.

6. To ensure sufficient Std1 to bind MIG2's promoter, we impose

$$\text{Std1}_{\text{tot}} = K_{\text{MIG2Std1}} + \theta_{\text{Std1}} \tag{12}$$

with θ_{Std1} positive.

7. To limit the effect of SNF1 on Mig1, we fit θ_{Mig1} rather then e_{Mig1}, where

$$e_{\text{Mig1}} = 10^{\theta_{\text{Mig1}}} \cdot i_{\text{Mig1}} \tag{13}$$

and θ_{Mig1} is positive and bounded.

8. To limit the effect of glucose on Mig2, we fit θ_{Mig2} rather then d_{Mig2g} where

$$d_{\text{Mig2g}} = 10^{\theta_{\text{Mig2}}} \cdot d_{\text{Mig2}} \tag{14}$$

where θ_{Mig2} is positive and bounded.

9. We expect more molecules of Mth1 compared to Std1 [31], and so fit θ_{Mth1} rather than s_{Mth1} where

$$s_{\text{Mth1}} = d_{\text{Mth1}} \left(\text{Std1}_{\text{tot}} + \theta_{\text{Mth1}}\right) \tag{15}$$

with θ_{Mth1} positive, so that the maximal amount of Mth1 obeys $s_{\text{Mth1}}/d_{\text{Mth1}} > \text{Std1}_{\text{tot}}$.

10. We expect Mth1 to regulate HXT4, and so fit θ rather than K_{HXT4Mth1} with

$$K_{\text{HXT4Mth1}} = \frac{s_{\text{Mth1}}}{d_{\text{Mth1}}} \cdot \mathrm{e}^{-\theta} \tag{16}$$

with θ positive, so that the maximal amount of Mth1 is greater than K_{HXT4Mth1}.

5.2.2 Simulating and fitting the model for Hxt4

We formulated the model using the Sencillo compiler, a Python version of Facile [36], and exported to both Julia and Python.

Simulating with Julia: We simulated the model in Julia (v1.11) using the OrdinaryDiffEq library (v6.89.0) with the TRBDF2 solver. TRBDF2 (Trapezoidal Rule with second-order Backward Differentiation Formula) is an implicit Runge-Kutta method that combines the trapezoidal rule and the second-order backward differentiation formula. This method is particularly suitable for stiff problems [37]. We set the absolute and relative tolerances to 10^{-6} (`abstol = 1e-6` and `reltol = 1e-6`). To incorporate time-varying glucose levels, we used linear interpolation to ensure a continuous glucose input.

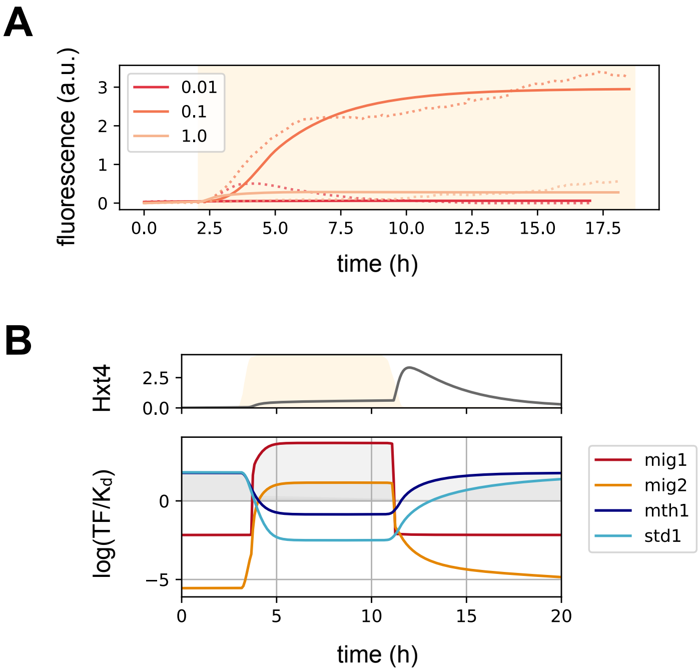

Fig E. The HXT4 model is valid for both types of glucose inputs shown in Fig 1. A The HXT4 model fits the data from Fig 1B, here shown as time series. We indicate glucose with shading. Solid lines show the model's predictions; dashed lines show the data. Glucose concentrations are in percentages (g/100mL). **B** The predicted behaviours of the transcription factors for a shift in and out of 0.4% glucose. Identically to Fig 2C, we plotted the $\log_2$ of the predicted level of repressors scaled by their inferred dissociation constants of binding to the HXT4 promoter.

Fitting the model: Let Y_{ij} be the measured fluorescence from condition i at time point j, M_{ij} be the model's predicted fluorescence, and W_i be the number of experimental replicates for condition i. Then our measure for the goodness of fit is

$$\sum_{ij} (Y_{ij} - M_{ij})^2 / (Y_{ij}/W_i) \tag{17}$$

We chose this measure to weigh the conditions by the available amount of experimental evidence and to reduce sensitivity to extreme values.

We set bounds on the parameters, omitting units for clarity:

1. All Hill numbers obeyed $1 < n, m < 5$.
2. All K parameters, those starting with K, obeyed $10^{-4} < K < 10^4$.
3. All d parameters, including d_{Mth1Snf3} and d_{Mth1Rgt2}, obeyed $10^{-4} < d < 100$.
4. All i parameters for nuclear entry obeyed $0.01 < i < 100$.
5. All e parameters for nuclear exit, including e_{Std1Rgt2} and e_{Std1Snf3}, obeyed $0.01 < e < 100$.
6. All s parameters for protein synthesis obeyed $0.01 < s < 100$.
7. For SNF1, $10^{-3} < L < 1000$.
8. We set $0.01 < \theta_{\mathrm{Mth1}} < 100$; $0.1 < \theta_{\mathrm{Std1}} < 100$; and $0.1 < \theta_{\mathrm{Rgt2Snf3}} < 10^4$.
9. All r parameters for intracellular glucose obeyed $0.1 < r < 100$.

To find sets of best-fit parameters efficiently, we used a Julia implementation of a black box optimiser (BlackBoxOptim), which uses a mixed strategy for fitting and does not require the goodness-of-fit function to be differentiable. We ran BlackBoxOptim using an exponential natural evolution (:xnes) method with a cutoff step size of 10^{-6}.

5.2.3 Optimal values of the parameters and initial conditions

The simulated behaviour of HXT4 with these optimal parameters matches well the experimental data (Fig 2B & Fig E).

For the values of the parameters, concentrations are dimensionless because we normalise fluorescence measurements by the fluorescence of Mig1-GFP; time is in hours.

SNF1:

$$\begin{array}{rcccrcl} \mathrm{SNF1_{tot}} & = & 1 & ; & L & = & 0.0054 \\ K_{\mathrm{SNF1Std1}} & = & 0.015 & ; & n_{\mathrm{SNF1}} & = & 1.0 \\ K_{\mathrm{SNF1}} & = & 0.001 & ; & m_{\mathrm{SNF1}} & = & 1.06 \end{array}$$

Hxt4:

$$\begin{array}{rcccrcl} s_{\mathrm{Hxt4}} & = & 10.66 & ; & d_{\mathrm{Hxt4}} & = & 0.167 \\ K_{\mathrm{HXT4Mig1}} & = & 0.045 & ; & n_{\mathrm{HXT4Mig1}} & = & 1.48 \\ K_{\mathrm{HXT4Mig2}} & = & 0.00029 & ; & n_{\mathrm{HXT4Mig2}} & = & 1.44 \\ K_{\mathrm{HXT4Mth1}} & = & 0.41 & ; & n_{\mathrm{HXT4Mth1}} & = & 5.0 \\ K_{\mathrm{HXT4Std1}} & = & 0.271 & ; & n_{\mathrm{HXT4Std1}} & = & 1.97 \\ K_{\mathrm{Hxt4g}} & = & 6460 & ; & m_{\mathrm{Hxt4}} & = & 4.85 \\ d_{\mathrm{Hxt4g}} & = & 0.192 & ; & & & \end{array}$$

Mig1:

$$\begin{array}{rcccrcl} i_{\mathrm{Mig1}} & = & 93.18 & ; & e_{\mathrm{Mig1}} & = & 93.18 \\ K_{\mathrm{Mig1SNF1}} & = & 0.0001 & ; & & & \end{array}$$

Mig2:

$$\begin{array}{rcccrcl} s_{\mathrm{Mig2}} & = & 0.011 & ; & d_{\mathrm{Mig2}} & = & 0.085 \\ K_{\mathrm{MIG2Mth1}} & = & 0.204 & ; & n_{\mathrm{MIG2Mth1}} & = & 1.49 \\ K_{\mathrm{MIG2Std1}} & = & 0.536 & ; & n_{\mathrm{MIG2Std1}} & = & 5.0 \\ K_{\mathrm{Mig2g}} & = & 0.096 & ; & m_{\mathrm{Mig2}} & = & 1.17 \\ d_{\mathrm{Mig2g}} & = & 47.17 & & & & \end{array}$$

Mth1:

$$\begin{array}{rcccrcl} s_{\mathrm{Mth1}} & = & 0.727 & ; & d_{\mathrm{Mth1}} & = & 0.516 \\ K_{\mathrm{MTH1Mig1}} & = & 1.52 & ; & n_{\mathrm{MTH1Mig1}} & = & 2.04 \\ K_{\mathrm{MTH1Mig2}} & = & 0.0022 & ; & n_{\mathrm{MTH1Mig2}} & = & 5.0 \\ K_{\mathrm{Snf3}} & = & 0.081 & ; & K_{\mathrm{Rgt2}} & = & 0.252 \\ d_{\mathrm{Mth1Snf3}} & = & 1.00 & ; & m_{\mathrm{Mth1Snf3}} & = & 4.98 \\ d_{\mathrm{Mth1Rgt2}} & = & 1.42 & ; & m_{\mathrm{Mth1Rgt2}} & = & 5.0 \end{array}$$

Std1:

$$\begin{array}{rcccrcl} \mathrm{Std1_{tot}} & = & 0.955 & ; & i_{\mathrm{Std1}} & = & 0.148 \\ e_{\mathrm{Std1Snf3}} & = & 0.98 & ; & m_{\mathrm{Std1Snf3}} & = & 4.87 \\ e_{\mathrm{Std1Rgt2}} & = & 2.02 & ; & m_{\mathrm{Std1Rgt2}} & = & 5.0 \end{array}$$

Intracellular glucose: For completeness, we give the optimal r parameters controlling the growth of intracellular glucose, although the simulations are insensitive to their exact values providing they are sufficiently large. For example, replacing $r_{\text{step}0.1}$ with a more feasible 98.0 given the other results generated time series visually indistinguishable from the fitted value of 5.2. Running simulations with $r_{\text{step}0.1} = 1$, however, did cause changes.

$$
\begin{array}{ccccccc}
r_{0.2} & = & 94.9 & ; & r_{0.4} & = & 98.7 \\
r_{1.0} & = & 96.6 & ; & r_{\text{step}0.01} & = & 92.4 \\
r_{\text{step}0.1} & = & 5.2 & ; & r_{\text{step}1.0} & = & 97.6 \\
r_{mig1\Delta 0.2} & = & 4.3 & ; & r_{mig1\Delta 0.4} & = & 98.5 \\
r_{mig1\Delta 1.0} & = & 95.5 & ; & r_{mth1\Delta 0.2} & = & 15.2 \\
r_{mth1\Delta 0.4} & = & 21.3 & ; & r_{mth1\Delta 1.0} & = & 100 \\
r_{rgt2\Delta 0.2} & = & 92.5 & ; & r_{rgt2\Delta 0.4} & = & 92.5 \\
r_{rgt2\Delta 1.0} & = & 88.9 & ; & r_{snf3\Delta 0.2} & = & 98.7 \\
r_{snf3\Delta 0.4} & = & 42.5 & ; & r_{snf3\Delta 1.0} & = & 93.3 \\
r_{std1\Delta 0.2} & = & 35.3 & ; & r_{std1\Delta 0.4} & = & 99.8 \\
r_{std1\Delta 1.0} & = & 92.2 & & & &
\end{array}
$$

Initial conditions: For the initial conditions, we first ran a simulation with zero glucose to steady state (`t=1.0e6`) starting with all initial concentrations at zero. We then replaced the initial value of Hxt4 with its measured value for the relevant experiment.

5.2.4 Parameter sensitivities

We calculated the parameter sensitivities (Fig F) using the eFAST (Extended Fourier Amplitude Sensitivity Test), which is conceptually similar to Sobol indices but computationally more efficient [38]. The first-order sensitivities measure the direct contribution of each parameter to how our goodness-of-fit function, Eq. 17, varies; the total-order sensitivities capture the full impact of each parameter, including any interactions with other parameters [39, 40].

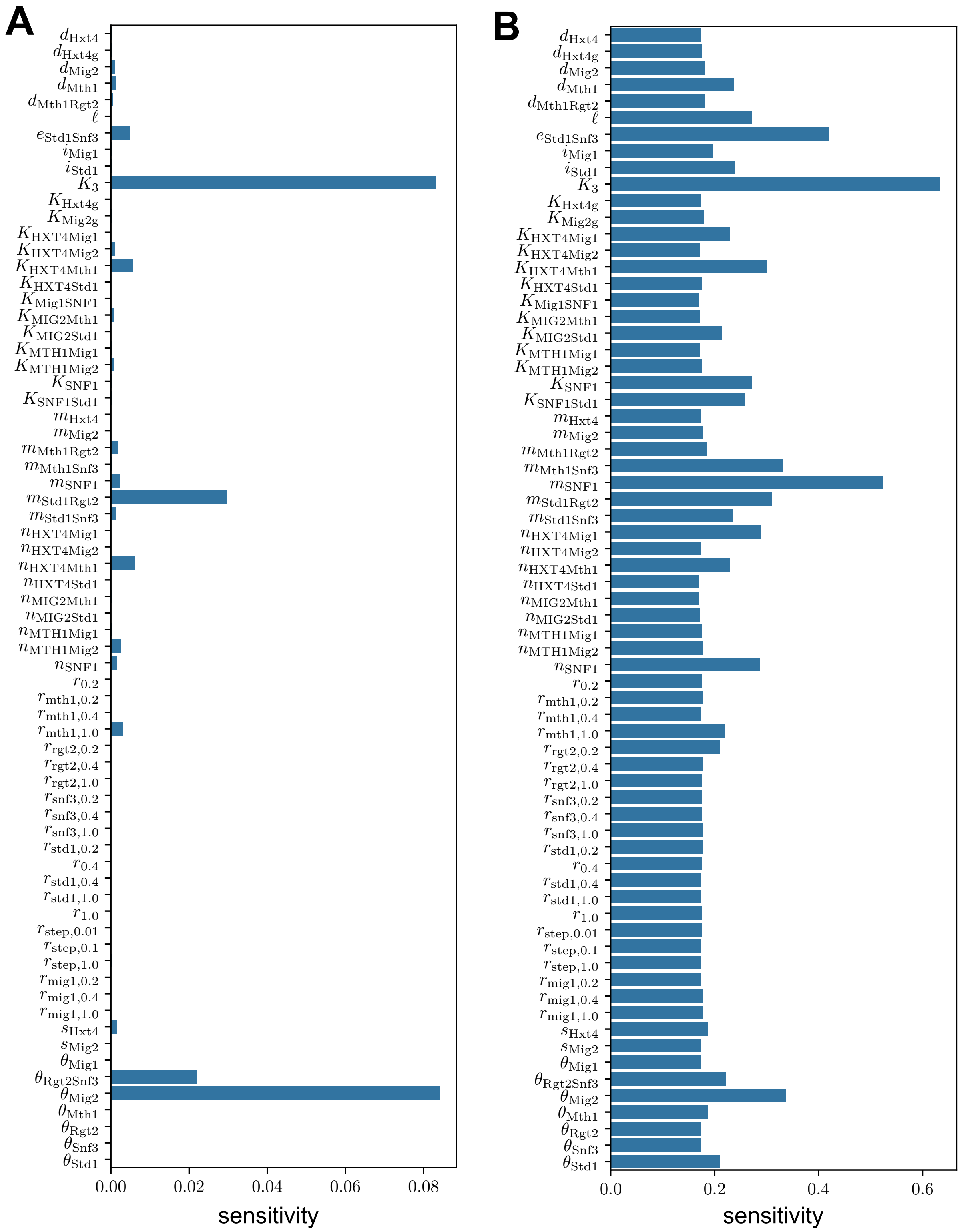

Fig F. The sensitivities of the HXT4 model's optimal parameters show the importance of the parameters controlling Mig2's degradation and the sensing of glucose. We used the eFAST and a perturbation window of size 10^{-5}. **A** The first-order sensitivities highlight the glucose affinities of Snf3 and Rgt2 and the glucose-induced degradation of Mig2. **B** The total-order sensitivities highlight in addition SNF1's response to glucose.

5.3 Fitting the other HXTs given HXT4's regulatory model

We used the regulatory model for HXT4, initially replacing Eq. 7 with an identical equation for the HXT under investigation. For each HXT, we ran an analysis using approximate Bayesian computation to determine which out of the possible promoter structures was most supported by Fig 1's data, changing the equivalent of Eq. 7 appropriately.

Determining prior distributions over promoter models: To generate prior probabilities on promoter structures, we used our plate-reader data [35] (Fig G).

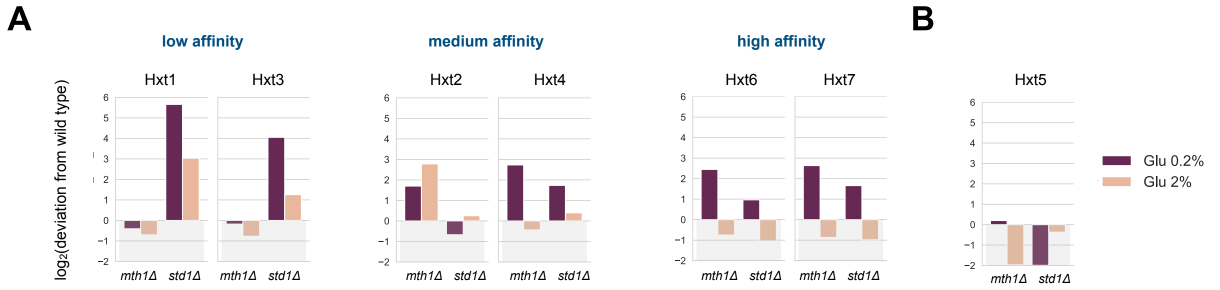

Fig G. The deviation of the Hxt-GFP fluorescence of deletion mutants from the wild-type fluorescence indicate whether Mth1 and Std1 regulate an HXT. Data are from plate readers [35]. We found the root mean square (RMS) of the difference in fluorescence at all time points between a deletion and the wild-type strain. We plot the logarithm of this RMS difference divided by the RMS difference between wild-type replicates. A positive value therefore indicates that a deletion strain's RMS is greater than that expected from measurement noise. **A** In the glucose concentrations measured, Std1 regulated the low-affinity transporters; Mth1 regulated HXT2; and Mth1 and Std1 both regulated HXT4, HXT6, and HXT7. **B** Neither Mth1 nor Std1 regulated HXT5.

We therefore assumed *a priori* that at least Std1 binds HXT1 and HXT3 and considered too promoters with all other combinations of repressors, that at least Mth1 binds HXT2 plus all other combinations, and that at least Mth1 and Std1 bind HXT6 and HXT7 plus all other combinations.

Implementing ABC in Julia: We developed an approach that provided in a single framework a comprehensive Bayesian treatment of both model uncertainty and parameter estimation. By incorporating different models following the ABC-SMC algorithm [41], we extended the Adaptive Population Monte Carlo (APMC) algorithm [42] to perform Bayesian model selection and simulation-based inference simultaneously. We initialised the algorithm by sampling from the prior distributions of all models. It then iteratively refined these samples by adjusting the acceptance threshold adaptively and resampling particles, continuing until reaching a specified minimum acceptance probability across all models. We thus ensured a thorough exploration of the joint space of models and parameters.

Our implementation in Julia used multithreading for efficiency, employed adaptive covariance estimation for proposal distributions, and incorporated a weighting scheme to correct for the bias introduced by the adaptive proposal [42].

Best fits: We show the fits of optimal models in Fig H and the predicted repression for each HXT in Fig I.

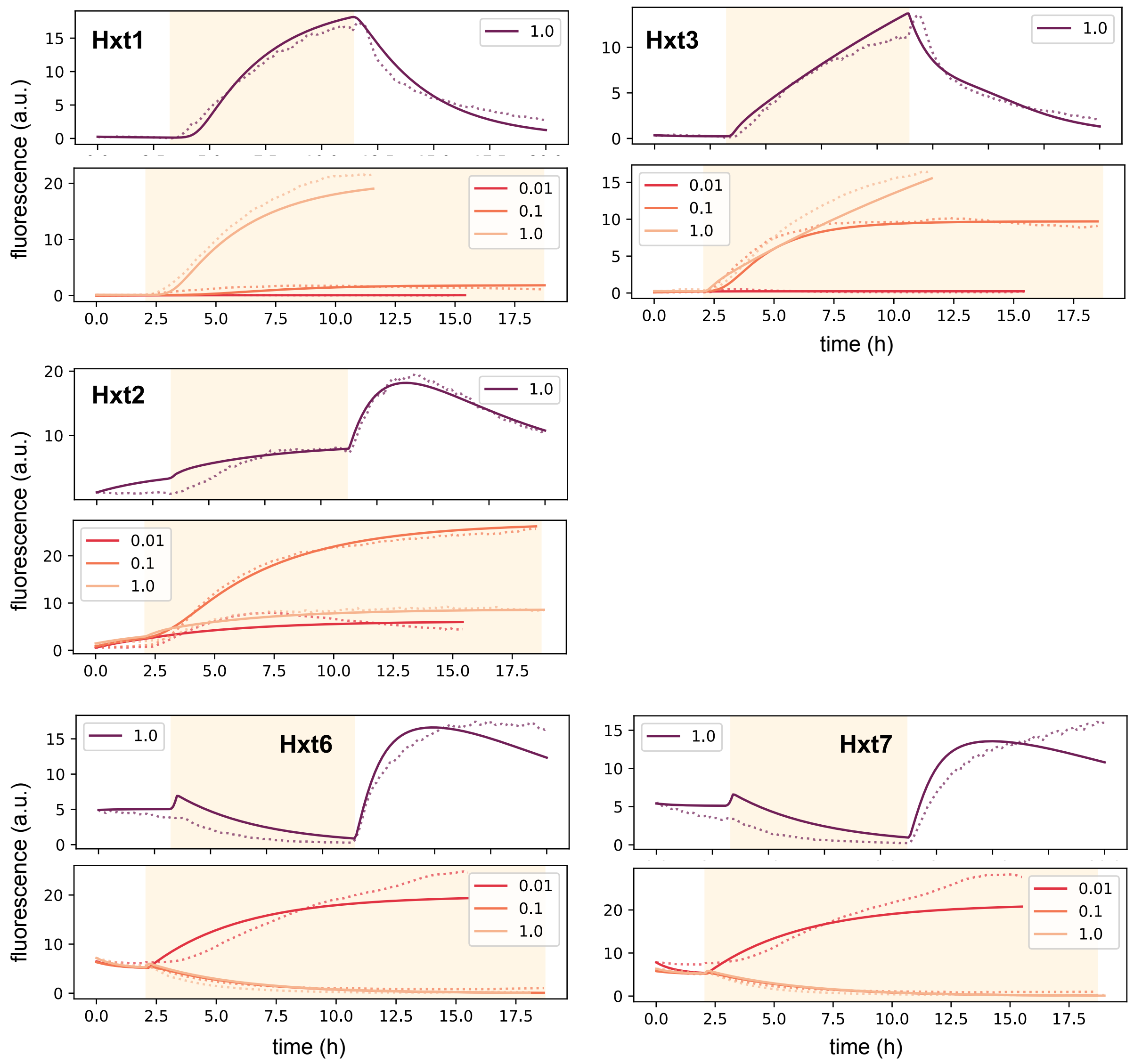

Fig H. The best-fit promoter models recovered the response for both types of glucose inputs shown in Fig 1. We plot the predicted behaviour for the most probable promoter model with its best-fit parameters as solid lines. Dashed lines show data. We use shading to indicate glucose and group Hxts by their affinity for glucose. If glucose concentrations differ from those shown here, we expect gradual rather than sharp changes in the responses: re-running the simulations for steps into constant glucose (bottom figures for each Hxt) for various concentrations of glucose between 0.005% and 2%, the responses smoothly altered from one concentration to the next.

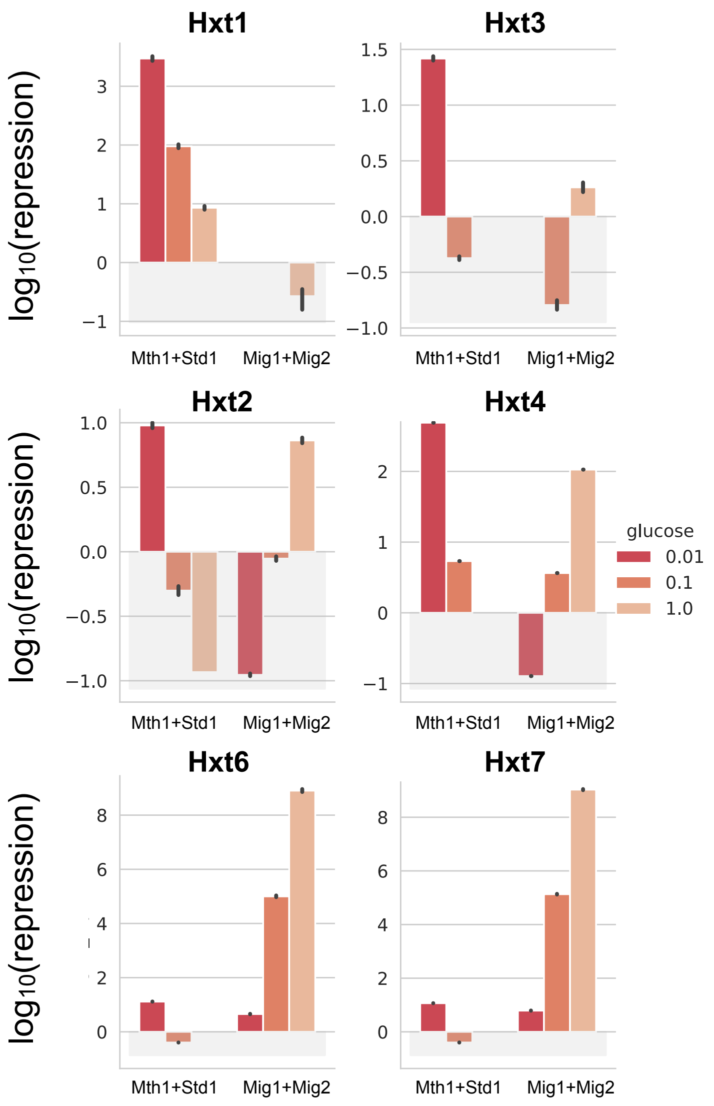

Fig I. The predicted repression for individual HXTs broadly follows the averaged behaviour of Fig 3C. We plot the logarithm of the predicted repression. The greater the repression is than one, the more it reduces transcription. Glucose concentrations are percentages (g/100 mL).

5.4 Caveats to the fitting

Neither our image-analysis pipeline nor our modelling distinguishes between Hxt-GFPs at the plasma membrane versus those in the vacuole (Fig A panel B), lumping both together. Not making this distinction may mean that the inferred degradation rates are underestimates because we erroneously considered any Hxts undergoing degradation in the vacuole as active.

6 Predicting transcriptional regulation from the promoter sequences

To investigate whether the sequences of the HXT promoters supported our predictions, we used SwissRegulon's predicted binding sites for transcription factors [43], specifically their predicted TF binding sites file (`sites.gff`) available at Downloads/Saccharomyces cerevisiae. These predictions use both weight matrices and the alignments of the intergenic regions of *S. cerevisiae* with those of four other *Saccharomyces* species [43]. We considered the promoter for each HXT

to be the DNA between the HXT's start site and the end of the next upstream gene. For each transcription factor and promoter, SwissRegulon gives potential binding sites and their posterior probabilities (Fig J).

For low concentrations of a transcription factor, the amount of factor bound at a site is proportional to its association constant of binding [44]. We assumed that this association constant is itself proportional to the site's posterior probability. We further assumed that for a given transcription factor at most only one binds to the promoter because of the low concentrations we considered. The mean occupancy of bound factor is then proportional to the sum of the posterior probabilities of all its binding sites in a promoter.

To find the occupancy of Rgt1, the transcription factor to which Std1 and Mth1 bind [45], we simply summed the posterior probabilities of its binding sites for each HXT promoter. For Mig1 and Mig2, all except one of the predicted Mig2-binding sites overlapped at least 60% with Mig1-binding sites. We therefore summed the posterior probabilities of only Mig1 sites, except for pHXT3 where we included too the posterior probability of the one unique Mig2 site. To display the extent of Rgt1 occupancy compared to Mig1 and Mig2's, we plotted the logarithm of their ratio (Fig 3D).

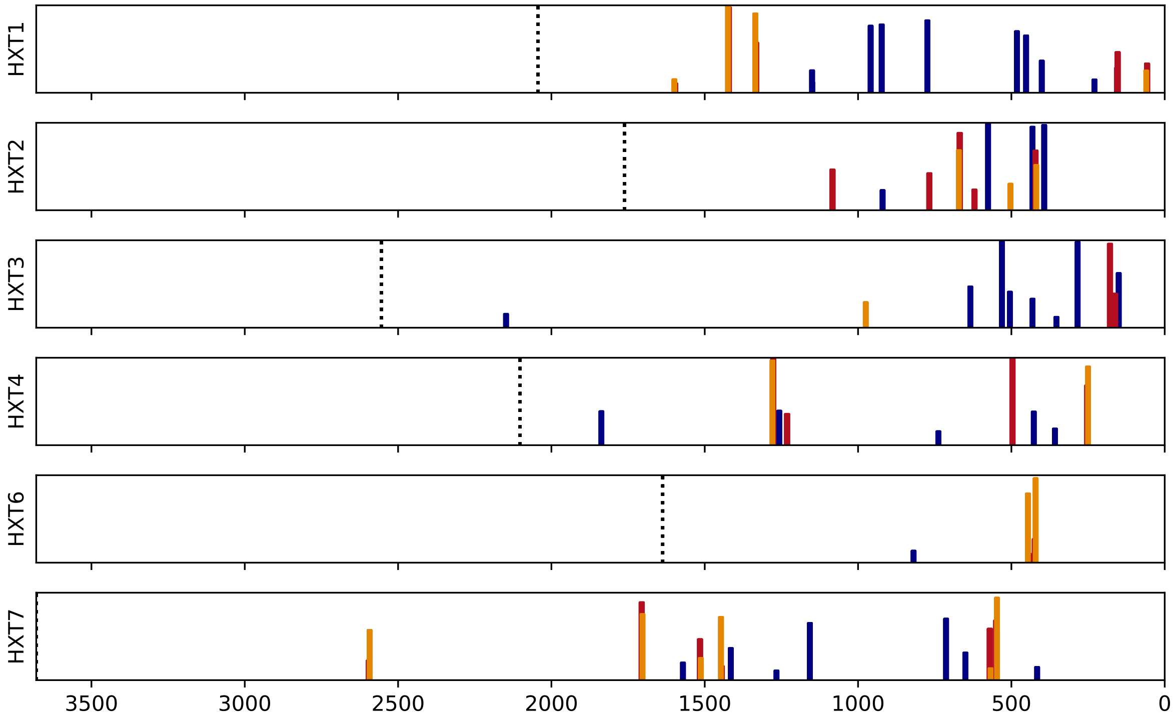

Fig J. Predicted binding sites for Rgt1, Mig1, and Mig2 for the HXT promoters regulated by the Snf3-Rgt2 network. Data are from SwissRegulon [43]. Rgt1 sites are in blue; Mig1 sites in red; and Mig2 sites in orange. The height of the bar at each site is proportional to its posterior probability. The x-axis shows the upstream, intergenic region for each gene and terminates at the dashed black line. The intergenic region for HXT7, the longest, is 3680 bases and that of HXT6, the smallest, is 1637 bases.

Comparison with other species of yeast

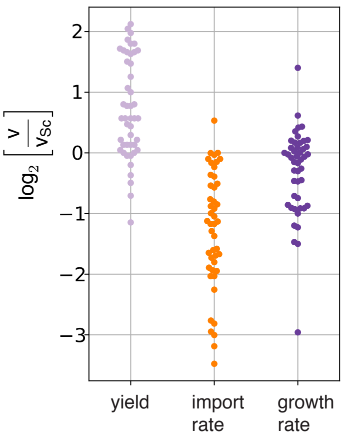

Fig K. Budding yeast excels at rapidly importing glucose. Comparing yield, the rate of glucose import, and growth rate across 46 species of yeast in 2% glucose indicates that budding yeast excels at importing glucose (data from [46]). We plot the $\log_2$ of the ratio of the growth characteristic, denoted v, to its value for budding yeast, denoted v_{Sc}. Negative results imply a lower value than budding yeast. Yield is the amount of biomass produced relative to the amount of glucose consumed. The only species to import at a greater rate than budding yeast is another *Saccharomyces* species – *S. mikatae*, which has 19 HXTs compared to *S. cerevisiae*'s 18 [47].

References

1. Sheff, M. A. & Thorn, K. S. Optimized cassettes for fluorescent protein tagging in Saccharomyces cerevisiae. *Yeast* **21,** 661–670 (2004).
2. Baudin, A., Ozier-Kalogeropoulos, O., Denouel, A., Lacroute, F. & Cullin, C. A simple and efficient method for direct gene deletion in Saccharomyces cerevisiae. *Nucleic Acids Res* **21,** 3329 (1993).
3. Longtine, M. S. *et al.* Additional modules for versatile and economical PCR-based gene deletion and modification in Saccharomyces cerevisiae. *Yeast* **14,** 953–961 (1998).
4. Youk, H. & Van Oudenaarden, A. Growth landscape formed by perception and import of glucose in yeast. *Nature* **462,** 875–879 (2009).
5. Janke, C. *et al.* A versatile toolbox for PCR-based tagging of yeast genes: new fluorescent proteins, more markers and promoter substitution cassettes. *Yeast* **21,** 947–962 (2004).
6. Tong, A. H. Y. & Boone, C. High-throughput strain construction and systematic synthetic lethal screening in Saccharomyces cerevisiae. *Met Microbiol* **36,** 369–707 (2007).
7. Engel, S. R. *et al.* The reference genome sequence of Saccharomyces cerevisiae: then and now. *G3–Genes Genom Genet* **4,** 389–398 (2014).
8. Lee, M. E., DeLoache, W. C., Cervantes, B. & Dueber, J. E. A highly characterized yeast toolkit for modular, multipart assembly. *ACS Synth Biol* **4,** 975–986 (2015).
9. Alam, M. T. *et al.* The metabolic background is a global player in Saccharomyces gene expression epistasis. *Nat Microbiol* **1,** 1–10 (2016).
10. Edelstein, A., Amodaj, N., Hoover, K., Vale, R. & Stuurman, N. Computer control of microscopes using μManager. *Curr Protoc Mol Biol* **92,** 14–20 (2010).

11. Crane, M. M., Clark, I. B., Bakker, E., Smith, S. & Swain, P. S. A microfluidic system for studying ageing and dynamic single-cell responses in budding yeast. *PLoS One* **9,** e100042 (2014).

12. Qin, D., Xia, Y. & Whitesides, G. M. Soft lithography for micro-and nanoscale patterning. *Nat Protocol* **5,** 491 (2010).

13. Granados, A. A. *et al.* Distributed and dynamic intracellular organization of extracellular information. *Proc Nat Acad Sci USA* **115,** 6088–6093 (2018).

14. Maier, A., Völker, B., Boles, E. & Fuhrmann, G. F. Characterisation of glucose transport in Saccharomyces cerevisiae with plasma membrane vesicles (countertransport) and intact cells (initial uptake) with single Hxt1, Hxt2, Hxt3, Hxt4, Hxt6, Hxt7 or Gal2 transporters. *FEMS Yeast Res* **2,** 539–550 (2002).

15. Reifenberger, E., Boles, E. & Ciriacy, M. Kinetic characterization of individual hexose transporters of Saccharomyces cerevisiae and their relation to the triggering mechanisms of glucose repression. *Eur J Biochem* **245,** 324–333 (1997).

16. Diderich, J. A., Merijn Schuurmans, J., Van Gaalen, M. C., Kruckeberg, A. L. & Van Dam, K. Functional analysis of the hexose transporter homologue HXT5 in Saccharomyces cerevisiae. *Yeast* **18,** 1515–1524 (2001).

17. Mi, H. *et al.* PANTHER version 16: a revised family classification, tree-based classification tool, enhancer regions and extensive API. *Nucleic Acids Res* **49,** D394–D403 (2021).

18. Katoh, K. & Standley, D. M. MAFFT multiple sequence alignment software version 7: improvements in performance and usability. *Mol Biol Evol* **30,** 772–780 (2013).

19. Nguyen, L.-T., Schmidt, H. A., Von Haeseler, A. & Minh, B. Q. IQ-TREE: a fast and effective stochastic algorithm for estimating maximum-likelihood phylogenies. *Mol Biol Evol* **32,** 268–274 (2015).

20. Bakker, E., Swain, P. S. & Crane, M. M. Morphologically constrained and data informed cell segmentation of budding yeast. *Bioinformatics* **34,** 88–96 (2017).

21. Swain, P. S. *et al.* Inferring time derivatives including cell growth rates using Gaussian processes. *Nat Commun* **7,** 1–8 (2016).

22. Broach, J. R. Nutritional control of growth and development in yeast. *Genetics* **192,** 73–105 (2012).

23. Einav, T., Mazutis, L. & Phillips, R. Statistical mechanics of allosteric enzymes. *J Phys Chem B* **120,** 6021–6037 (2016).

24. Hubbard, E., Jiang, R. & Carlson, M. Dosage-dependent modulation of glucose repression by MSN3 (STD1) in Saccharomyces cerevisiae. *Mol Cell Biol* **14,** 1972–1978 (1994).

25. Tomás-Cobos, L. & Sanz, P. Active Snf1 protein kinase inhibits expression of the Saccharomyces cerevisiae HXT1 glucose transporter gene. *Biochem J* **368,** 657 (2002).

26. Simpson-Lavy, K., Xu, T., Johnston, M. & Kupiec, M. The Std1 activator of the Snf1/AMPK kinase controls glucose response in yeast by a regulated protein aggregation. *Mol Cell* **68,** 1120–1133 (2017).

27. Treitel, M. A., Kuchin, S. & Carlson, M. Snf1 protein kinase regulates phosphorylation of the Mig1 repressor in Saccharomyces cerevisiae. *Mol Cell Biol* **18,** 6273–6280 (1998).

28. Kaniak, A., Xue, Z., Macool, D., Kim, J.-H. & Johnston, M. Regulatory network connecting two glucose signal transduction pathways in Saccharomyces cerevisiae. *Eukaryot Cell* **3,** 221–231 (2004).

29. Snowdon, C. & Johnston, M. A novel role for yeast casein kinases in glucose sensing and signaling. *Mol Biol Cell* **27,** 3369–3375 (2016).

30. Kim, J.-H., Brachet, V., Moriya, H. & Johnston, M. Integration of transcriptional and posttranslational regulation in a glucose signal transduction pathway in Saccharomyces cerevisiae. *Eukaryot Cell* **5,** 167–173 (2006).

31. Sabina, J. & Johnston, M. Asymmetric signal transduction through paralogs that comprise a genetic switch for sugar sensing in Saccharomyces cerevisiae. *J Biol Chem* **284,** 29635–29643 (2009).

32. Hovsepian, J. *et al.* Multilevel regulation of an α-arrestin by glucose depletion controls hexose transporter endocytosis. *J Cell Biol* **216,** 1811–1831 (2017).

33. Ho, B., Baryshnikova, A. & Brown, G. W. Unification of protein abundance datasets yields a quantitative Saccharomyces cerevisiae proteome. *Cell Syst* **6,** 192–205 (2018).

34. Ozcan, S., Dover, J., Rosenwald, A. G., Wölfl, S. & Johnston, M. Two glucose transporters in Saccharomyces cerevisiae are glucose sensors that generate a signal for induction of gene expression. *Proc Nat Acad Sci USA* **93,** 12428–12432 (1996).

35. Montaño-Gutierrez, L. F. *et al.* Analysing and meta-analysing time-series data of microbial growth and gene expression from plate readers. *PLoS Comput Biol* **18,** e1010138 (2022).

36. Siso-Nadal, F., Ollivier, J. F. & Swain, P. S. Facile: a command-line network compiler for systems biology. *BMC Syst Biol* **1,** 1–6 (2007).

37. Hosea, M. & Shampine, L. Analysis and implementation of TR-BDF2. *Appl Numer Math* **20,** 21–37 (1996).

38. Saltelli, A., Tarantola, S. & Chan, K.-S. A quantitative model-independent method for global sensitivity analysis of model output. *Technometrics* **41,** 39–56 (1999).

39. Sobol, I. M., Tarantola, S., Gatelli, D., Kucherenko, S. S. & Mauntz, W. Estimating the approximation error when fixing unessential factors in global sensitivity analysis. *Reliab Eng Syst Saf* **92,** 957–960 (2007).

40. Saltelli, A. *et al.* Variance based sensitivity analysis of model output. Design and estimator for the total sensitivity index. *Comput Phys Commun* **181,** 259–270 (2010).

41. Toni, T., Welch, D., Strelkowa, N., Ipsen, A. & Stumpf, M. P. Approximate Bayesian computation scheme for parameter inference and model selection in dynamical systems. *Interface* **6,** 187–202 (2009).

42. Lenormand, M., Jabot, F. & Deffuant, G. Adaptive approximate Bayesian computation for complex models. *Comput Stat* **28,** 2777–2796 (2013).

43. Pachkov, M., Balwierz, P. J., Arnold, P., Ozonov, E. & Van Nimwegen, E. SwissRegulon, a database of genome-wide annotations of regulatory sites: recent updates. *Nucleic Acids Res* **41,** D214–D220 (2012).

44. Van Nimwegen, E. Finding regulatory elements and regulatory motifs: a general probabilistic framework. *BMC Bioinformatics* **8,** S4 (2007).

45. Lakshmanan, J., Mosley, A. L. & Özcan, S. Repression of transcription by Rgt1 in the absence of glucose requires Std1 and Mth1. *Curr Genet* **44,** 19–25 (2003).

46. Hagman, A., Säll, T., Compagno, C. & Piskur, J. Yeast "make-accumulate-consume" life strategy evolved as a multi-step process that predates the whole genome duplication. *PLoS One* **8,** e68734 (2013).

47. Lin, Z. & Li, W.-H. Expansion of hexose transporter genes was associated with the evolution of aerobic fermentation in yeasts. *Mol Biol Evol* **28,** 131–142 (2011).
